# MYTHOS: A Python Interface for Surface Crystal Structure Prediction of Organic Semiconductors

**DOI:** 10.1021/acs.jcim.5c00669

**Published:** 2025-07-09

**Authors:** Emilio Lorini, Karsten Walzer, Martin Pfeiffer, Luca Muccioli

**Affiliations:** † Department of Industrial Chemistry, 9296 University of Bologna, Via Piero Gobetti, 85, 40129 Bologna, Italy; ‡ 367886 Heliatek GmbH, Treidlerstraße 3, 01139 Dresden, Germany

## Abstract

We introduce a new computational approach for predicting organic crystalline structures on flat surfaces, an essential step in designing and optimizing thin-film systems for electronic devices. Based on molecular mechanics and molecular dynamics simulations, and implemented in a user-friendly Python program, this method enables a sequential layer-by-layer analysis of crystalline formation, thus allowing to identify surface-induced polymorphs (SIPs) and to study the transition between surface and bulk structures. A validation against six diverse test cases demonstrated a good match with experimental crystalline parameters and arrangements, underscoring the reliability of the method in identifying the most relevant polymorphs for a given molecule.

## Graphical abstract

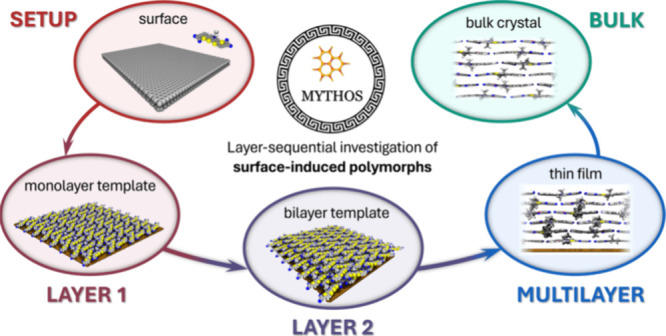

## Introduction

Organic semiconductors (OSCs) represent a diverse group of molecules that offer valuable advantages over traditional inorganic semiconductors. Notably, they exhibit mechanical flexibility, cost-effective fabrication, and a significant tuneability of their optoelectronic properties, making them particularly suitable for electronic applications. These benefits are especially relevant in the realization of devices such as light-emitting diodes (OLEDs),^1^ organic solar cells (OSCs),^2^ and organic field-effect transistors (OFETs),^3^ in which the precise molecular arrangement of the semiconducting material is directly linked to its functional properties and to device efficiency.^4^ Therefore, understanding and controlling molecular growth upon fabrication can give important insights toward enhancing device performances. Typical manufacturing techniques include vapor phase deposition and spin coating, which are performed on a substrate that allows the formation of thin film structures.^5,6^ Similarly to single crystal growth in solution, thin film growth is strongly influenced by molecular interactions, kinetic and thermodynamic conditions, but it is further affected by molecule–substrate interactions over the resulting molecular packing and orientation. The growth mechanism might be then influenced to some extent by the surface morphology and yield a morphology different from that observed in bulk single crystals. When organic molecules bind strongly to a crystalline substrate, crystals might grow through full epitaxy,^7,8^ but even in absence of epitaxy and notably also on amorphous substrates, vapor-deposited molecules still can produce other structures than the ones observed in bulk environment, the so-called substrate-induced polymorphs (SIPs).^9,10^ Depending on the specific molecule and substrate, SIPs may extend until a certain critical thickness, typically hundreds of nm, after which the bulk crystal structure is restored.^11^ The simultaneous presence of crystalline domains with diverse morphologies has a direct effect on the overall electronic properties:^12^ regardless of their nature (i.e., bulk or substrate), different polymorphs typically exhibit very different charge carrier mobilities, as found in the case of pentacene^13,14^ and rubrene.^15,16^ For this reason, controlling polymorphism in thin films and tuning manufacturing techniques toward the occurrence of highly conducting crystalline forms is essential for performance optimization.^17^

The preliminary investigation of potential crystalline structures for new molecules can enhance the specificity of molecular design and consequently alleviate the number of attempted synthesis processes, which are frequently costly and laborious. Crystal structure prediction (CSP) is an ever-evolving field that might help in this context. While CSP has spread out toward the development of many different approaches over the years, it is generally conceived as a multistage method that progressively selects and refines the most stable crystalline organizations. CSP encloses and blends several computational techniques, such as Monte Carlo Simulated Annealing (MCSA) for structure candidate generation, periodic density functional theory (pDFT) and force field (FF) calculations for energy evaluation, and lattice dynamics (LD) or biased molecular dynamics (MD) simulations for free energy evaluation.^18,19^ Machine learning was also recently implemented for the optimization of stages like energy revaluation from FF level to DFT^20^ or for the total prediction of interatomic potentials,^21^ a key strategy employed by some of the best performing groups during the last CSP blind test, a contest periodically held by the Cambridge Crystallographic Data Center (CCDC).^22^

Despite major progress involving CSP for bulk crystals, we are currently not aware of any method specifically designed to predict crystalline structures grown in thin film and the occurrence of possible SIPs. A valid alternative is to perform simulations of vapor deposition,^23–28^ which however only reveal the most probable polymorph under the given kinetic and thermodynamic conditions and are often computationally expensive, limiting their practical application for this purpose.

In this paper, we present a new and inexpensive polymorph screening methodology that allows Surface Crystal Structure Prediction (SCSP) in thin films. The main idea behind SCSP is the sequential identification of the lowest energy molecular organizations at increasing number of layers placed on a given surface. This approach allows for a first screening of the most stable 2-dimensional organizations found on the surface and subsequent analysis of the 3-dimensional one, which is naturally influenced by the former. Our method is implemented in a semiautomated program, MYTHOS (Morphological surveY for THin-films of Organic Semiconductors), that employs MD simulations for the generation of random molecular aggregates on a flat surface and FF energy evaluations for stability classification.

MYTHOS was first tested over three test case molecules whose crystalline characterization in bulk and on-surface has been extensively studied: pentacene, perfluoropentacene and α-sexithiophene (Figure 1). Despite the structural similarity, pentacene (PEN) owns an opposite charge distribution compared to perfluoropentacene (PFP), which makes them p-type and n-type respectively, employable together in heterojunction structures.^29^ In addition to charge transport properties, the two molecules differ also with respect to their crystal structure on certain substrates. Indeed, while they both present a herringbone disposition in the most stable polymorphs found in single crystals,^30–32^ when grown on strongly interacting flat surfaces (e.g., graphite), PFP crystals are flat-lying on the surface,^33^ while PEN crystals still present the same molecular organization observed in bulk.^34^ α-sexithiophene (α-6T) is another p-type carrier that was widely studied and used in transistors.^35^ A similar crystalline tendency is found between PEN and α-6T, which single crystals are characterized by a herringbone disposition in both LT and HT polymorphs.^36,37^ When growing on a strongly interacting substrate, α-6T can organize in a row-arranged flat-lying disposition, as found for monolayers on flat Au surfaces.^38,39^ The method was also tested over three newly synthesized dicyanovinyl derivatives, specifically designed for OSC applications: DCV5T-Me_2_,^40^ DCV-Fu-PyT-Fu-iPr,^41,42^ and DCV-T-TPyT-T-Pr.^43^ For clarity, these molecules will be referred to in this paper as DCV-1, DCV-2, and DCV-3, respectively (corresponding IUPAC names are reported in SI). Their chemical structures share a common acceptor–donor–acceptor (A–D–A) framework, featuring an electron-rich core of condensed or conjugated heterocycles, flanked by electron-deficient dicyanovinyl terminations. This architecture makes them highly effective photoabsorbers due to their reduced optical gaps and enhanced optical properties.^44,45^ Notably, these molecules exhibit a distinct crystalline thin-film arrangement, aligning parallel to the substrate and enabling 3D growth driven by π-stacking interactions, as observed experimentally. This behavior is attributed to the stabilization provided by hydrogen bonding between cyano groups and the hydrogen atoms of the conjugated core. Another key design feature is the inclusion of alkyl moieties of variable length attached to the electron-rich core: several studies have demonstrated that both the position and length of these chains significantly influence the 3D molecular packing and, consequently, the overall performance of the device.^40,46,47^

**Figure 1:**
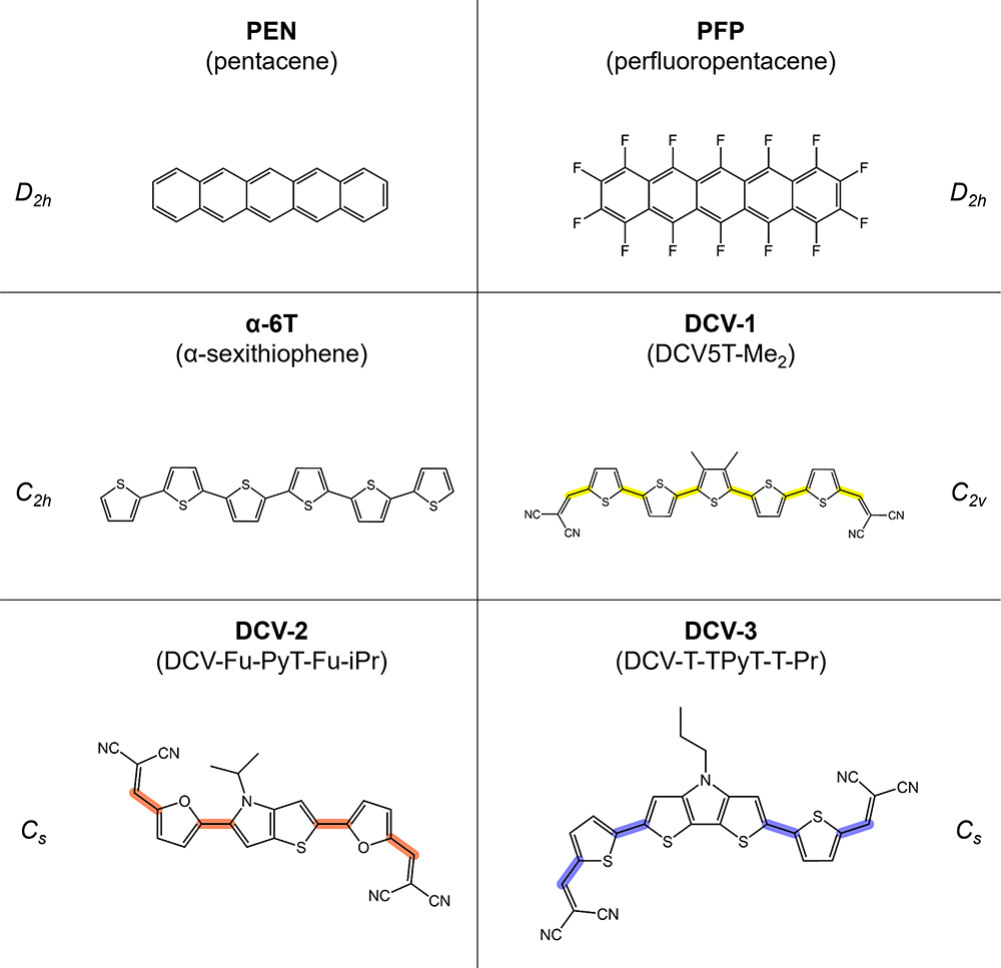
Chemical structures of the test molecules investigated with MYTHOS: pentacene, perfluoropentacene, α-sexithiophene (all-*trans* conformation) and the conformers found in the crystalline structures of DCV-1, DCV-2, and DCV-3. The torsion angles with highlighted central bonds were blocked during the simulations.

Recently, Ortmann and co-workers carried out a computational study on 2-dimensional crystal structure prediction for coplanar arrangements of molecules similar to the ones treated here.^48^ However, the study was not extended to multilayer formation and the possible effects of bulk relaxation on the quasi-2D arrangements, while we show here that monolayers with coplanar molecular arrangements sometimes turn into staggered configurations upon relaxation of multilayer structures. Moreover, even if the approach in reference^48^ has the merit of including a systematic conformational search and ranks polymorphs with DFT-D level accuracy, it is computationally more expensive (of several orders of magnitude, depending on the system size) and does not take into account the chemical nature and the morphology of the surface. Conversely, in the following, it is shown how MYTHOS effectively identifies both planar and 3D crystal structures for most reported polymorphs of the test molecules including specific interactions with the surface.

## Computational Methodology

MYTHOS workflow can be summarized into a minimum of five sequential steps, depending on the number of layers singularly investigated, as sketched in Figure 2:

a) *Molecule setup* (SET)
b) *First monolayer investigation* (LAY1)
c) *Construction of the first monolayer template* (BUILD1)
d) *Second monolayer investigation* (LAY2)
e) *Optional X^th^* *monolayer investigation:*
  - *Construction of the [X–1]* *^th^* *layers template* (BUILD­[X-1])
  - *X* *^th^* *monolayer investigation* (LAYX)
f) *Multilayer investigation* (MULTI)

**Figure 2:**
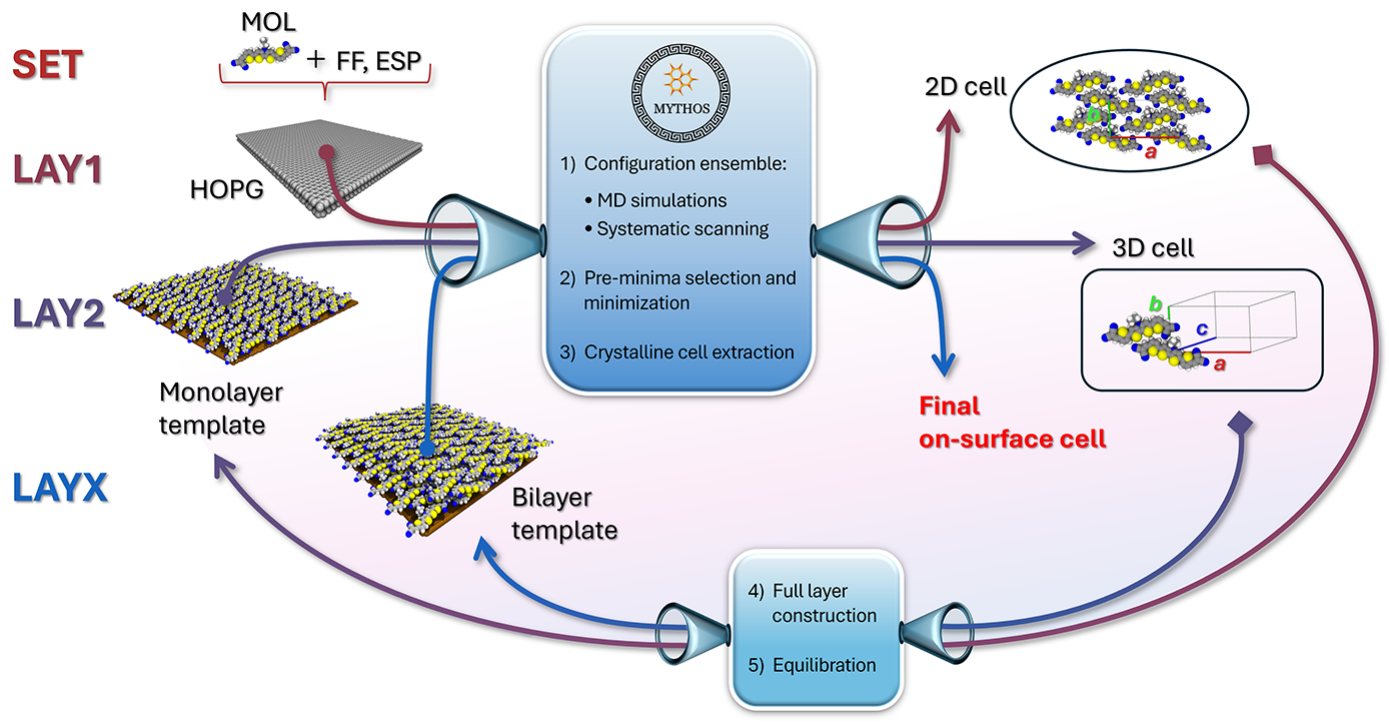
Scheme of MYTHOS workflow. Within the blue frames are listed the computational steps performed for each investigated layer.

Each step is based on either minimization or molecular dynamics simulations performed with the software NAMD^49^ using periodic boundary conditions (PBCs) in all directions. When a surface is present in the system, a value of 400 Å is set for the *z* direction to simulate the vacuum, while *x*,*y* sides depend on the size of the specific surface. Atoms of any surface were kept frozen to their initial position in all simulations discussed in the article, although for the LAY1 step the user can opt for allowing the motion of surface atoms and the ensuing thermalization of the surface. The Particle Mesh Ewald (PME) method^50^ is used to compute the Coulomb interactions across PBCs, using a cutoff of 12 Å for the calculation in the direct space, as well as for truncating Lennard–Jones interactions. Temperature is kept constant by rescaling the atomic velocities every 100 steps. Simulations are performed with constant volume and cell dimensions, excluding the ones performed at constant pressure of 1 atm with a Berendsen barostat. Minimizations are carried out with the conjugated gradient method and in absence of constraints.

### (a) Molecule Setup (SET)

MYTHOS performs morphological predictions of single conformers of organic molecules. A force field is built automatically for the molecule of interest, extracting geometrical and Lennard-Jones parameters from the General Amber Force Field (GAFF)^51^ while Coulomb atomic charges are calculated externally with a QM software (Gaussian16 ^52^ or ORCA^53^) with a level of theory of choice. Specifically, the program recognizes element and connectivity of each atom and assigns atom types and corresponding potential energy terms. If the chosen conformer is subjected to unwanted interconversion around specific rotational bonds during the dynamics, it is possible to constrain any torsion angle to a chosen value and prevent this structural change. Alternatively, external topology and parameter files in CHARMM format can be provided to the program and used in the simulations.

Before the investigation of the layer morphology, it is then required to choose the molecular conformer of study. For flexible molecules and in absence of any a priori information, a conformational study is recommended before MYTHOS usage, e.g., with the popular CREST software like proposed in ref 48. It is important to note that the lowest-energy conformer of a single molecule is not necessarily the one found in a crystal, as the conformer choice influences factors such as packing density. Especially if two or more conformers have similar energy, it may be unavoidable to carry out steps (b) to (f) for all of them to identify the most plausible packing. Once the conformer is chosen, the user must delve into the molecular symmetry to search for two types of symmetry elements: rotation axes parallel to the molecular plane (the molecule is supposed to be lath-shaped) or orthogonal planes to these axes. If the symmetry group does not include any of these elements, the morphological analysis for each layer should be subdivided into two branches: whether including only one molecular face (Single Face, SF) or both faces at the same time (Double Face, DF) in the system. Indeed, the two faces of the molecule would not be symmetrically equivalent once placed on a flat surface like HOPG (i.e., they are enantiomers in a two-dimensional space), since the molecular flip is hampered by the interactions with the substrate.

### (b) First Monolayer Investigation (LAY1)

The aim of this step is to explore a vast ensemble of diverse planar configurations of molecular aggregates on top of a given surface and guess a 2D crystalline cell from those at lowest energy. In this work we chose a surface of Highly Ordered Pyrolytic Graphite (HOPG), which is flat and chemically similar to the organic species of study, for which π-stacking interactions are frequently relevant. The default surface (88.416 × 106.350 Å^2^) was built by replicating 36 × 25 times the orthorhombic HOPG unit cell (*a* = 2.456 Å, *b* = 4.254 Å, *c* = 6.696 Å) along the *ab* surface axes. Alternatively, the user can build a specific HOPG surface indicating the desired dimensions and number of layers, or any other preconstructed surface, as long as it is relatively flat and chemical reactions with the target molecules can be ruled out.

Two types of approaches are available to produce a collection of independent system configurations of the target molecules on the surface:

1. *MD simulation of free molecules*: A selected number of molecules is let free to diffuse over the graphite surface for a chosen simulation time in the order of nanoseconds. High temperatures guarantee a larger morphological variety; the downside is the possibility of desorption of the molecules from the surface (which depends on the adsorption energy) or possible unwanted conformational changes.
  Simulations with a low number of molecules (2–4) can give important insights about the specific first neighbor interactions but lack of a realistic aggregation effect given by the presence of many neighbors. Instead, simulating a larger number of molecules (5–8) could produce a larger variety of aggregates which, however, can have a rather disordered and irregular 2D structure.
2. *Scanning of position and orientation of planar aggregates*: A first aggregate (or single molecule) is positioned near a second planar aggregate or molecule, placed on the graphite surface with fixed coordinates. The aggregate could either be obtained from option 1 or from previous scans performed for a smaller number of molecules. The configurations are generated systematically by iteration over all the combinations of a chosen set of orientations and positions with respect to the second aggregate. This option benefits from the higher maneuverability in the morphological exploration and is computationally faster, due to the absence of MD simulations. However, the kinetic energy contribution and entropic effects are missing in the generation of possible stable organizations.

Regardless of the generation method, the molecular configurations are then sorted according to the intermolecular energy within the molecular aggregates (*E*_inter_), calculated as follows:

$$E_{\mathrm{inter}}=E^{\mathrm{TOT}}-E_{\mathrm{intra}}=E^{\mathrm{TOT}}-\sum_{i}^{N}E_{i,\mathrm{intra}}$$ 1

where *E* ^TOT^ is the total potential of the molecules (i.e., excluding the surface contributions) and *E*_intra_ is the intramolecular energy of all molecules, given by the sum of the potential energy of each isolated molecule *E*_*i*_.

The ensemble of configurations is filtered to exclude consecutive trajectory frames and configurations with low degree of molecular aggregation. Specifically for the latter, a user-defined reference atom is chosen for each molecule. Two molecules are considered neighbors if the distance between their reference atoms is smaller than a predefined cutoff. An adjacency matrix is constructed for molecular pairs, and isolated molecular clusters are excluded through power analysis of the matrix.

A minimization takes place for a chosen number of the filtered configurations at lowest energy (preminima) and the potential of interaction *E*_int_ is calculated again. The stability order of the minima may change after the minimization: the most stable minimum may not correspond to the most stable preminimum. Finally, the most stable minima are arranged into classes based on structural similarity: all the interatomic distances within the aggregate are calculated and compared one-to-one between all minima. If the RMSD between two aggregates is smaller than a chosen threshold, they are assigned to the same class.

The planar crystalline parameters (*a*, *b*, γ) can be extracted from the most stable minimized configuration (MSMCs) of a chosen class. This operation is possible when the aggregate presents at least three molecules with the same orientation on the surface, thus allowing to individuate the conventional **a** and **b** vectors between the first and second molecule and first and third molecule respectively (Figure 3a).

**Figure 3:**
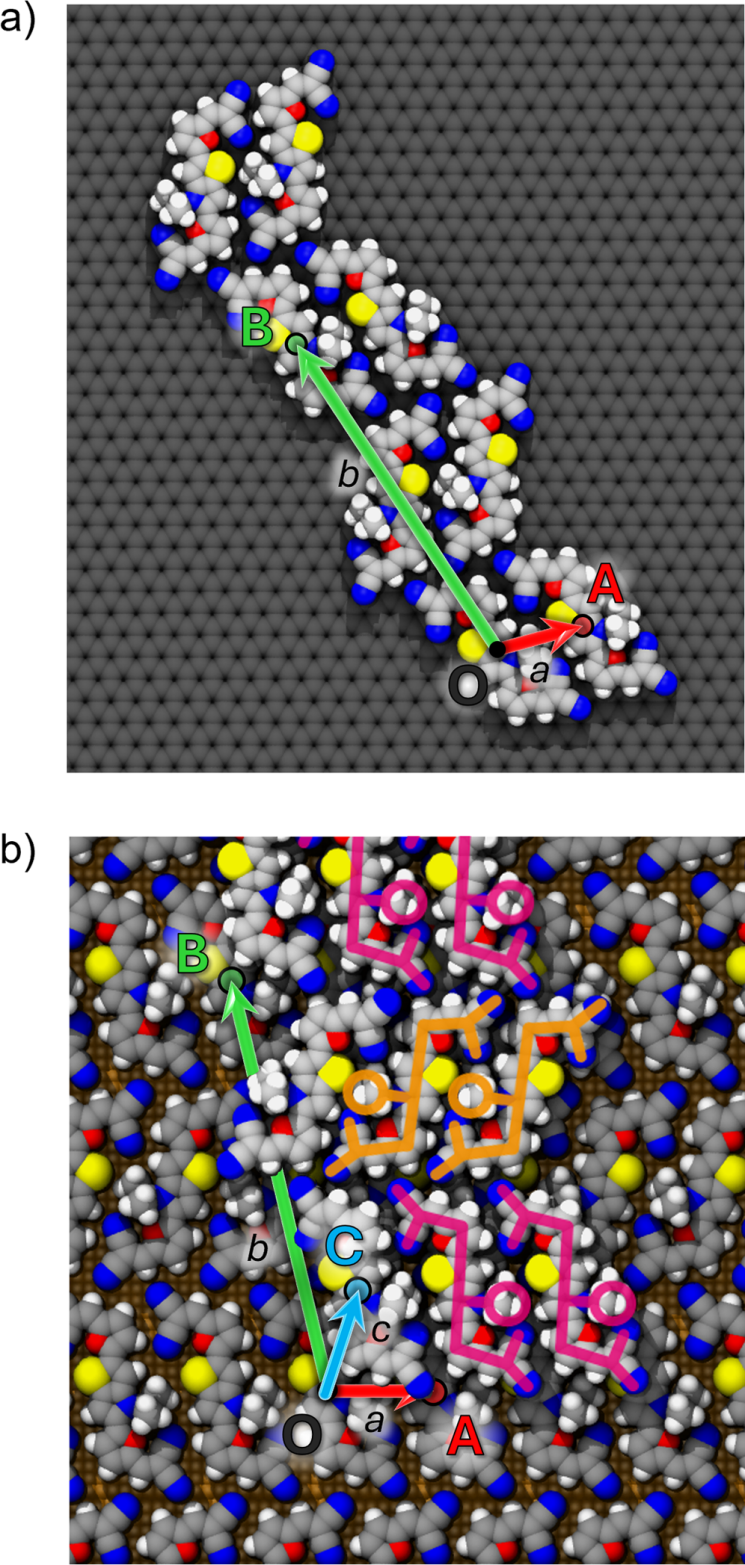
Example of lattice parameters extraction in a 2D DCV-2 aggregate placed on a HOPG surface (a) and a crystalline aggregate placed on top of the first monolayer on the GLS substrate (b). Lattice vectors (**a**, **b**, **c**) correspond to the distance vectors between the centers of mass (colored circles) of molecules with the same orientation (O, A, B, C). In the presence of molecules with different orientation, the number of formula units Z in the 2D cell is higher than 1 (*Z* = 2 in this depiction).

MYTHOS allows cell calculation according to any triclinic convention. In this paper, crystalline cells are defined assigning **a** and **b** vectors to the short and long sides parallel to the surface plane respectively, while **c** is the out-of-plane vector. This convention is a logical choice within the MYTHOS framework, which first focuses on the 2D crystalline morphology (**ab**) and then considers the third vector for 3D morphologies (**c**).

### (c) Construction of the First Monolayer Template

Planar unit cells can be replicated along *a* and *b* to build a monolayer template to be placed on the same HOPG surface used at step (b). However, there might be a relative mismatch between the dimensions of the monolayer, restricted to integer multiples of its unit cell with rather large *ab* dimensions and that of graphite supercell (with integer multiples of *a* = 2.456 Å, *b* = 4.254 Å). Minimizing this mismatch would possibly require a significant increase in the size of the HOPG surface, which can quickly become computationally prohibitive. Additionally, the free space stemming from this mismatch could, in some cases, facilitate molecular horizontal motion leading to a subsequent deviation from the starting planar morphology. Therefore, to minimize the mismatch, it is convenient to replace the graphite with an artificial flat substrate with a smaller unit cell, made of equidistant dummy atoms placed on the intersections of a square grid with mesh size of 1 Å, dubbed “graphite-like square” (GLS) substrate, that can obviously be adapted to the dimensions of the first monolayer replica within 1 Å of margin. The Lennard-Jones parameters of the dummy atoms can be adjusted to reproduce a similar energetic landscape to that of the graphite surface or any other flat surface (Figure S1), or more in general they can be modified to modulate the strength of the molecule–surface interaction.

During step (c), the first monolayer is positioned close to the substrate at a height determined by the user. The new system can be either minimized or equilibrated at a certain temperature. In the second case, a short equilibration (50 ps) allows the adsorption of the layer on the substrate, according to the intermolecular interactions, and a subsequent longer equilibration (>1 ns) is performed to relax the system avoiding the drift of the total center of mass over the surface. After this step, the full monolayer equilibrated (or minimized) on the GLS substrate is ready to be employed as a template for the investigation of the second monolayer morphology.

### (d) Second Monolayer Investigation (LAY2)

The procedure is similar to the one explained at step (b): a large number of different configurations is generated for molecules placed on top of a complete first monolayer, from which the most stable ones are selected and minimized prior to the spatial unit cell extraction.

At this step, three approaches are possible to generate the configuration ensemble:

1. *MD simulation of free molecules on the molecular template:* The procedure is identical to that on graphite, described at step (b) point (1).
2. *MD simulation of a constrained aggregate on the molecular template:* A molecular aggregate obtained from the planar unit cell is placed on top of a complete first monolayer. During the dynamics, the aggregate moves as a whole on the first layer surface thanks to the application of harmonic potentials on the interatomic distances between the molecules.
3. *Scanning of position and orientation of an aggregate atop the template*: Similarly to the lateral scanning for the first layer (step b) point (2), the configurations are obtained from the systematic iteration over a set of positions and orientation of a planar aggregate on top of the first monolayer.

Before sorting the molecular configurations, two types of energy quantities are evaluated. The first is the intermolecular potential between the molecules in the second layer (*E*_inter_^*L*2–*L*2^):

$$E_{\mathrm{inter}}^{L2-L2}=E^{L2}-E_{\mathrm{intra}}^{L2}=E^{L2}-\sum_{i}^{N}E_{i,\mathrm{intra}}^{L2}$$ 2

where *E* ^L2^ is the total potential of the molecules in the second layer and *E*_intra_^*L*2^ is the intramolecular potential of all molecules in the second layer, given by the sum of the intramolecular potential energy of each molecule *E*_*i*,intra_^*L*2^. Also, the intermolecular potential between the molecules of the second layer and those of the first layer (*E*_inter_^*L*2–*L*1^) is calculated from the total interaction potential of the molecules in the second layer (*E*_inter_^*L*2–*TOT*^) as follows:

$$E_{\mathrm{inter}}^{L2-\mathrm{TOT}}=E^{\mathrm{TOT}}-E^{L1}-E_{\mathrm{intra}}^{L2}$$ 3

$$E_{\mathrm{inter}}^{L2-L1}=E_{\mathrm{inter}}^{L2-\mathrm{TOT}}-E_{\mathrm{inter}}^{L2-L2}$$ 4

where *E* ^TOT^ is the total potential of the molecules (excluding the surface contributions) and *E* ^*L*1^ is the total potential of the molecules in the first layer.

As done for LAY1 step, the produced configurations are sorted according to their energy (here the sum of *E*_inter_^*L*2–*L*2^ and *E*_inter_^*L*2–*L*1^) and the most stable ones are minimized and grouped into classes based on the geometry of the aggregates of second layer only. In this way, second layer aggregates of the same class, but with different shift respect to the first layer, are directly compared to reveal which shifts produce the lowest energy.

The whole set of crystalline parameters (*a*, *b*, *c*, α, β, γ) is then derived from the minimized crystalline structures of a specific class through the user-specified selection of at least four molecules with equivalent orientation (i.e., with the same alignment with respect to the substrate axes), one of which is found in the upper layer, to define the **a**, **b**, **c** vectors (Figure 3b). These molecules must belong to the same packing motif and be arranged in a periodic fashion to exhibit minimal rotational deviation from each other. The cell stability is subsequently evaluated through the calculation of the cohesive energy per molecule *E* ^COH^ on a minimized supercell of cubic-like dimensions (≃150 molecules) without applications of constraints, through the following equation:

$$E^{\mathrm{COH}}=\frac{1}{Z}\sum_{i}^{Z}(E^{\mathrm{TOT}}-E_{i}^{\mathrm{TOT}-\mathrm{MOL}}-E_{i}^{\mathrm{MOL}})$$ 5

where *E* ^TOT^ is the energy of the supercell, *E*_*i*_^TOT–MOL^ is the energy of the supercell without the *i*th molecule and *E*_*i*_^MOL^ is the energy of the isolated *i*th molecule. The operation is performed for each of the *Z* molecules in the unit cell and averaged to account for the contribution of possible asymmetric units.

### (e) Optional *X* ^th^ Monolayer Investigation

In the case of occurrence of different molecular orientations in the first and second layer, the extraction of the final on-surface cell is not possible in the sole presence of the first two layers and the investigation should continue for of a third or more layers (BUILD­[X-1], LAYX), which is still possible within the same framework followed for the second layer calculations.

### (f) Multilayer Investigation

Once the 3D unit cell is obtained, it is possible to perform further simulations in the presence of a higher number of complete layers. The crystalline parameters and the type of aggregation can be confirmed and the results become more general and comparable with experimental quantities. Indeed, experimental samples of OSCs obtained through vapor deposition contain many stacked layers and the effect of the surface gradually decreases over the number of deposited molecules. For this reason, multilayer calculations can be performed in two types of environments: either on the surface with a higher number of layers, or in bulk simulations to neglect the surface entirely (Figure 4).

**Figure 4:**
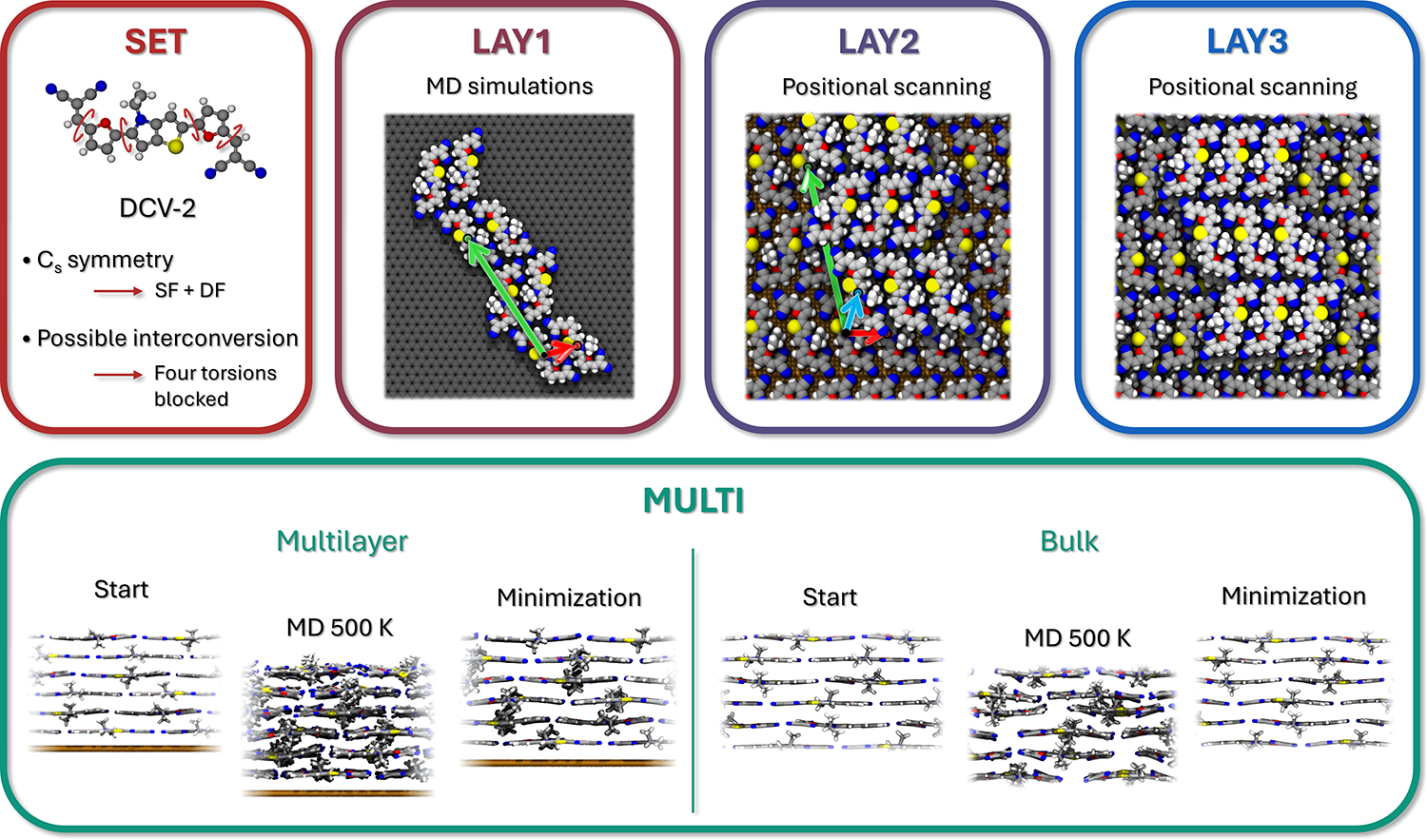
Scheme employed for the identification of the crystal cell of a family of aggregates (“class C”) found for the DCV-2 molecule. The identification of ***a***, ***b*** cell vectors (red and green, respectively) is illustrated for molecules with the same orientation in a 2D aggregate placed on a HOPG surface (LAY1) and then the three **a**, **b**, ***c*** cell vectors (red, green, and cyan) in a 3D crystalline aggregate placed on the GLS substrate (LAY2). Simulations at LAY3 were necessary for further refinement of the lattice parameters before being employed for the calculation of both on-surface and bulk crystalline cells. Additional depictions of the crystalline classes at each layer are provided in the Supporting Information for each test molecule.

#### On Surface

A multilayer system is obtained from the replica of the unit cell and placed on the artificial substrate. The system is equilibrated in the canonical ensemble (NVT) conditions for at least 1 ns at a chosen temperature, for instance the one used during vapor depositions or that employed during crystallographic measurements. The refined unit cell is then extracted from the molecules in the center of the multilayer systems, to minimize the influence of the surface at the bottom and of the absence of interactions with the vacuum at the top.

#### Bulk Environment

The final unit cell of the crystal on the surface is used to create a supercell of a relatively high number of molecules in all three directions and then simulated in the bulk using 3D PBCs at a chosen temperature. Three simulations are performed in sequence: a short NVT step (200 ps), a longer step in the isobaric–isothermal ensemble (NPT) (typically more than 1 ns) and a final minimization. The NVT step is meant for preliminary thermalization. Instead, during the NPT step, the volume of the supercell is allowed to change, and the box sides and angles can shrink or enlarge according to the intermolecular interactions within the system. Once the system has reached thermal equilibrium, the unit cell in bulk environment can be extracted and compared with that obtained in the presence of the surface. From this comparison it can be inferred if the absence/presence of the surface is actually relevant in producing a different morphology, i.e., if the polymorph obtained on the surface is a free energy minimum also in the bulk.

## Discussion

Having described the general computational framework, in the following we discuss the outcome of the prediction of the crystalline structures of the six test molecules represented in Figure 1, while the description of the specific methodological details for each case is left to the Supporting Information. The experimental crystalline parameters reported in Tables 1–6 were recalculated according to MYTHOS convention and the relative cohesive energy was determined upon minimization using the same force field as SCSP, applied to a supercell generated by replicating the experimental structure.

**Table 1:** Experimental and Predicted Crystalline Cells of PEN Molecules Grown On-Surface and in Bulk: Lattice Sides (Å), Angles (°), Molecular Units per Cell (*Z*), Volume per Molecule (nm^3^), and Cohesion Energy per Molecule (kcal/mol)^a^

			*a*	*b*	*c*	α	β	γ	*Z*	*V*	*E* ^COH^
on-surface	Exp	^34^	6.3	14.8				89.6			
	Simul	Dep^27^	6.2	16.1	8.9	127.2	84.6	82.0	2	0.350	–69.8
		SCSP	6.0	16.2	8.5	126.6	94.6	82.2	2	0.327	–63.9
bulk	Exp	LT^30^	6.1	16.0	7.9	112.6	85.8	101.9	2	0.346	–73.6
		HT^31^	6.1	15.1	8.1	80.9	85.9	76.7	2	0.357	–71.7
	Simul	SCSP	6.1	16.2	8.0	112.1	85.0	109.9	2	0.346	–65.6

a The unit cell extracted from vapor deposition simulation on graphite is reported as well.

### Pentacene

During LAY1 step, pentacene molecules in all minimized configurations were found to be parallel to the graphite surface with their aromatic plane (the most stable aggregate is shown in Figure S2a. However, a morphological change occurred for multilayer systems where molecules were found still horizontal but in a herringbone type of organization. While the extent of this change is feeble during LAY2 step (Figure S2b), with molecules still flat-lying in the first layer and acquiring a small tilt component with respect to the substrate plane in the second, the herringbone configuration becomes more evident from simulations of a four-layer system (Figure S3b). Interestingly, defects and cavities started to form within the multilayers upon these simulations, due to the different area occupation of the flat-lying and herringbone configurations. Indeed, while the former tends to fully occupy the horizontal space parallel to the substrate plane, the onset of herringbone packing increases the vertical space occupation. The subsequent availability of a wider horizontal free space causes a higher freedom for horizontal molecular motion and a consequent increase of disorder. Nevertheless, crystalline regions are still present within the system and a unit cell can be extracted. This morphological evolution with increasing number of layers is in agreement with the one observed experimentally during the growth of pentacene crystals on graphite^34^ and also the crystalline parameters of the final structure reflect this correspondence (Table 1). In addition, when removing the substrate and performing a simulation in bulk, the final crystal structure becomes very similar to the polymorph characterized by Campbell et al.,^30^ indicating that the surface polymorph is actually induced by the flat substrate.

### Perfluoropentacene

Unlike their pentacene analogues, PFP molecules were found to form stable aggregates with a flat-lying disposition both in single and multilayer systems in which herringbone packing was never observed. Such morphology and crystalline parameters (Table 2) are in agreement with the results obtained from XRD characterization during the growth of PFP crystals on flat substrates.^33^ Starting from this structure, PFP molecules retain their planar organization even after 5 ns-long bulk simulations at different temperatures (300–700 K). We note that experiments on bulk PFP crystals indicate instead a herringbone-like molecular organization,^32^ that is as well retained in MD simulations starting from this latter morphology. The similar stability of the two polymorphs (Table 2) shows how the phase transition from the planar to the herringbone structure is not thermodynamically favored, at least for the employed force field, and might be also hampered by a high energetic barrier.

**Table 2:** Experimental and Predicted Crystalline Cells of PFP Molecules Grown On-Surface and in Bulk: Lattice Sides (Å), Angles (°), Molecular Units per Cell (*Z*), Volume per Molecule (nm^3^), and Cohesion Energy per Molecule (kcal/mol)^a^

			*a*	*b*	*c*	α	β	γ	*Z*	*V*	*E* ^COH^
on-surface	Exp	^33^	8.9	15.1	6.5	108.1	78.6	92.4	2	0.410	–77.4
	Simul	Dep^27^	9.1	15.5	7.1	109.8	74.5	91.7	2	0.451	–76.4
		SCSP	8.4	17.3	7.5	124.4	79.3	102.8	2	0.438	–77.0
bulk	Exp	^32^	4.7	15.7	11.7	91.6	90.0	90.0	2	0.399	–76.7
	Simul	SCSP	8.4	17.4	7.7	122.2	100.2	103.0	2	0.462	–74.4

a The unit cell extracted from vapor deposition simulation on graphite is reported as well.

### α-Sexithiophene

We evaluated α-sexithiophene (α-6T) crystallinity for the all-*trans* conformer depicted in Figure 1, which is found in the polymorphs observed experimentally.^36,37^ The all-*trans* structure belongs to the C_2h_ symmetry group, which does not include any rotation axes on the molecular plane. Therefore, we performed two branches of on-surface calculations: whether including in the system only molecules lying on one molecular “face” (Single Face, SF) or allowing both faces (Double Face, DF). Similarly to PEN, α-6T molecules tend to organize in a flat-lying disposition in the first monolayer and only once upper layers are introduced, they start to acquire a tilt angle. Three types of polymorphs were found during the layer investigation: A_1_ and A_2_, presenting only one face in each monolayer, with A_2_ exhibiting alternating faces in successive layers, and B, which contains both faces already in the first monolayer. Four full layers of each class (Figure S4d) were built, and the final on-surface crystalline cell was extracted (Table 3). Seen the absence of measured 3D crystalline parameters for α-6T crystals on flat surfaces, we are able to compare only the in-plane parameters (*a*, *b*, γ), which are compatible with those obtained for a crystalline monolayer grown on Au(001) surfaces.^38^ Interestingly, from simulations in bulk environment we could observe the formation of a distinct herringbone organization for each class of surface polymorph, which are close in energy. In particular, the morphology of A_1_ is similar to the LT polymorph, with one face in the plane parallel to the main molecular axis, while A_2_ resembles the HT polymorph, with both faces in the plane parallel to the molecular axis.

**Table 3:** Experimental and Predicted Crystalline Cells of α-6T Molecules Grown On-Surface and in Bulk: Lattice Sides (Å), Angles (°), Molecular Units per Cell (*Z*), Volume per Molecule (nm^3^), and Cohesion Energy per Molecule (kcal/mol)

			*a*	*b*	*c*	α	β	γ	*Z*	*V*	*E* ^COH^
on-surface	Exp	^38^	6.7	25.2				98.2	1		
	SCSP	A_1_	6.4	26.1	7.8	53.7	92.1	86.0	2	0.522	–102.9
		A_2_	6.4	26.0	7.5	59.0	90.1	85.5	2	0.527	–102.8
		B	15.1	23.4	10.6	40.0	65.5	78.4	4	0.537	–94.0
bulk	Exp	LT^36^ 500 K	6.0	50.5	7.8	90.0	90.0	62.3	4	0.544	–106.0
		HT^37^ 580 K	6.0	26.4	8.4	54.5	89.9	89.8	2	0.539	–105.6
	SCSP	A_1_	6.3	26.2	8.2	55.6	94.9	86.8	2	0.543	–100.7
		A_2_	6.0	25.9	7.8	59.7	92.4	89.7	2	0.522	–107.0
		B	14.7	23.5	10.8	41.8	66.7	78.7	4	0.564	–97.1

### DCV-1

X-ray measurements of DCV-1 single crystals reveal that molecules adopt distinct orientations within each layer, arranged in a staggered configuration. Notably, a face-to-face interaction is observed between layers for molecules with opposite orientations, i.e., rotated 180° around the orthogonal axis.^40^ MYTHOS SCSP calculations for the first monolayer identified two main energetically favorable configurations, A and B, both exhibiting the two orientations observed experimentally but differing on the magnitude of lattice vector **a**. Instead, A and B structures show a very similar pattern along the lattice vector **b**, where the two orientations (in yellow and gray in Figure 5a) alternate. Compared to other organizations, this type of architecture allows for the formation of a well-defined hydrogen bonding network, established between the dicyanovinyl moieties and the hydrogens of the conjugated core. LAY2 investigation showed that each class of 2D polymorphs may present in turn two possible organizations, differing in the positional shift of the upper molecules with respect to the bottom ones with the same orientation: A_1_/B_1_, with smaller horizontal shift (*c* ≃ 10 Å), and A_2_/B_2_, with larger shift (*c* ≃ 20 Å) as shown in Figure 5b. Upon multilayer simulations performed at 500 K, we observed how molecules deviate from planarity and the two dicyanovinyl units bend in opposite directions as happening in the reference cell measured experimentally.^40^ In addition, always in the multilayer system, we noticed the occurrence of a deviation from the planar layer arrangement, resulting in a tilted configuration. This structural change gives rise to the formation of staggered layers once the surface is removed (Figure S5), i.e., during bulk simulations of A_1_, A_2_, and B_2_. Among those, the bulk B_2_ simulation result in a structure with crystalline parameters closely aligned with the bulk experimental cell (Table 4).

**Figure 5:**
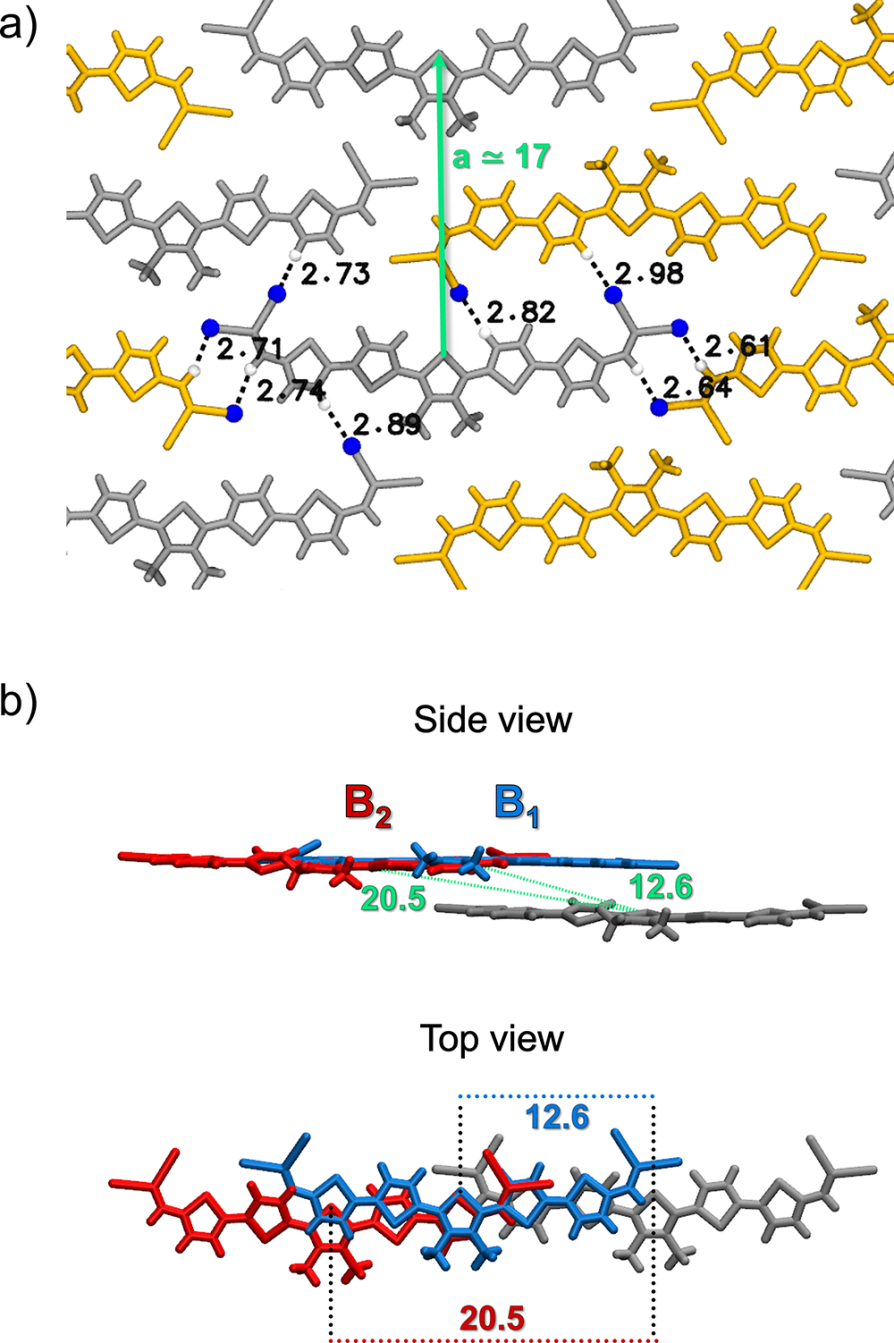
Representation of B polymorphs of DCV-1: (a) 2D molecular arrangement showing two distinct orientations (gray and orange) and forming a hydrogen bond network and (b) 3D organization of B_1_ and B_2_ polymorphs, highlighting the difference in positional shift between the first and second layer along the **c** vector. Distance values are reported in Ångstrom.

**Table 4:** Experimental and Predicted Crystalline Cells of DCV-1 Molecules Grown On-Surface and in Bulk: Lattice Sides (Å), Angles (°), Molecular Units per Cell (Z), Volume per Molecule (nm^3^), and Cohesion Energy per Molecule (kcal/mol)

			*a*	*b*	*c*	α	β	γ	*Z*	*V*	*E* ^COH^
on-surface	SCSP	A_1_	26.8	51.5	9.6	158.2	51.8	140.8	4	0.700	–140.0
		A_2_	27.1	51.5	20.1	170.5	38.4	139.8	4	0.719	–129.5
		B_1_	17.3	51.6	12.6	164.4	85.0	94.1	4	0.751	–135.3
		B_2_	17.2	52.0	20.4	170.5	90.8	90.7	4	0.735	–136.5
bulk	Exp	^40^	15.9	50.5	20.1	170.2	90.0	90.0	4	0.684	–144.8
	SCSP	A_1_	26.8	51.4	9.6	158.4	51.7	140.8	4	0.698	–140.1
		A_2_	27.3	51.2	20.1	170.0	37.5	139.2	4	0.726	–137.5
		B_1_	17.3	51.6	12.6	164.4	85.0	94.1	4	0.751	–136.1
		B_2_	17.2	52.1	20.5	170.5	91.6	90.9	4	0.735	–139.0

### DCV-2

Like in the α-6T case, the low symmetry of DCV-2 (and DCV-3) requires considering two possible dispositions on the surface (“faces”) and then conducting both SF and DF calculations. During the LAY1 investigation we identified three primary organizations (A, B, and C). The first two configurations present only one molecular face in the first monolayer but differ for the presence of either two different orientations (A) or a single orientation (B) within the monolayer, hence they have one and two formula units in the 2D cell, respectively. The third configuration (C) was obtained from DF calculations and exhibited both faces within the first monolayer, meaning again *Z* = 2. The second layer presents the same configuration as the first but rotated by 180° around the *z* axis. Accordingly, the total number or units in the 3D cell becomes 4. Despite significant differences in their planar organization, all the corresponding 3D structures were stabilized by a shifted arrangement between layers. Together with a slight deviation from planarity, this positional shift between layers facilitates the interlayer insertion of the iso-propyl chain, as observed in the XRD structure.^48^ The hydrogens at the conjugated core remain closer to the nitrogen atoms than the aliphatic ones (Figure S9), making them more likely to form hydrogen bonds. The proximity of a nitrogen atom to multiple hydrogens, both aromatic belonging to molecules of the same layer and aliphatic from molecules in neighboring layers, suggests potential multiple H-bond interactions. Notably, the crystalline parameters of class B match very closely those of the experimental cell for both on-surface and bulk crystal simulations, as detailed in Table 5. To evaluate the sensitivity of the prediction to force field parameters, SCSP of DCV-2 was performed also using the Dreiding force field,^54^ following a specific reparameterization process,^55^ also employed in reference.^56^ The comparison (Table S2) shows that both force fields recover similar low-energy crystalline cells, with relatively minor differences in lattice parameters and cohesive energies, with variation of a few percents in cell parameters and energies.

**Table 5:** Experimental and Predicted Crystalline Cells of DCV-2 Molecules Grown On-Surface and in Bulk: Lattice Sides (Å), Angles (°), Molecular Units per Cell (*Z*), Volume per Molecule (nm^3^), and Cohesion Energy per Molecule (kcal/mol)

			*a*	*b*	*c*	α	β	γ	*Z*	*V*	*E* ^COH^
on-surface	SCSP	A	8.9	34.5	9.3	25.3	107.7	95.1	2	0.515	–115.7
		B	8.9	17.0	7.0	91.6	90.4	83.7	2	0.523	–117.4
		C	8.8	34.7	13.4	31.3	88.9	88.6	4	0.532	–120.6
bulk	Exp	^48^	8.7	17.5	6.8	90.0	90.0	90.8	2	0.520	–125.1
	SCSP	A	8.8	34.3	11.1	23.3	106.3	96.6	2	0.593	–123.9
		B	8.9	16.9	7.2	76.9	89.6	84.1	2	0.525	–128.9
		C	8.9	34.7	13.3	31.5	89.9	89.3	4	0.536	–127.0

### DCV-3

The DCV-3 conformer found from XRD measurements of single crystals^48^ belongs to *C*_*s*_ symmetry group, and its two molecular faces are not equivalent once placed on a surface. The experimental crystalline structure comprises molecules of both faces, in staggered layers organized in a face-to-face configuration with a small horizontal shift (*xy* shift, i.e., **c** vector). Due to this peculiar disposition, hollow pockets are found between dicyanovinyl moieties of neighboring molecules with the same face and are occupied by the propyl alkyl chains of the underlayer molecules of the other face. Such intricate packing, with the propyl alkyl chain leaning out from the same face in all molecules, is favored by a slight deviation from molecular planarity, which allow for a larger extension of the alkyl chains in space and for the formation of weak hydrogen bonds between cyano nitrogens and the alkyl hydrogen atoms of the two terminal carbons.

Owing to the higher number of degrees of freedom of DCV-3, caused by a higher structural and chemical complexity compared to the acenes for instance, MD simulations of aggregates on HOPG did not show a significant tendency toward the formation of long-living clusters. Positional scans allowed instead the identification of stable planar aggregates with the propyl chain leaning out-of-plane. Two main classes of configurations were identified according to the presence of one (A) or two faces (B) within the planar unit cell. From the investigation of the organization in the upper layers, we could also detect two stable polymorphs for each planar class, differing in the positional shift of the molecules in the second layer respect to the ones of bottom layer, similarly to what was found for DCV-1. Therefore, a total of four 3D crystalline cells were found (Figure S7): A_1_/B_1_ with small face-to-face shift and *Z* = 2, and A_2_/B_2_ with a larger shift and *Z* = 2, see Table 6.

**Table 6:** Experimental and Predicted Crystalline Cells of DCV-3 Molecules Grown On-Surface and in Bulk: Lattice Sides (Å), Angles (°), Molecular Units per Cell (Z), Volume per Molecule (nm^3^), and Cohesion Energy per Molecule (kcal/mol)

			*a*	*b*	*c*	α	β	γ	*Z*	*V*	*E* ^COH^
on-surface	SCSP	A_1_	9.6	26.9	5.6	140.9	115.7	47.0	1	0.655	–127.0
		A_2_	9.5	27.0	10.4	152.7	112.2	46.8	1	0.647	–125.5
		B_1_	14.5	26.6	5.3	138.1	86.5	91.3	2	0.678	–122.9
		B_2_	14.9	26.5	13.3	150.1	62.9	92.3	2	0.692	–112.9
bulk	Exp	^48^	13.1	26.7	4.3	125.4	90.0	90.0	2	0.618	–141.0
	SCSP	A_1_	9.7	27.0	5.7	142.8	117.7	47.5	1	0.657	–136.9
		A_2_	9.7	28.7	7.17	98.7	92.2	44.9	2	0.697	–128.1
		B_1_	14.5	26.6	5.3	138.1	86.5	91.3	2	0.678	–126.9
		B_2_	14.9	26.5	13.3	150.1	62.9	92.3	2	0.692	–122.1

Upon bulk NPT simulations, molecules reorganize and start to deviate from the original coplanar structure in order to optimally occupy the space. When starting from A_1_ and B_1_ structures, molecules assume a staggered configuration within the layers, similarly to the one observed experimentally. This structural transition is caused by the tendency of bulky propyl chains to insert within the cavities of the lower or upper layer, which was previously hampered by the presence of the flat substrate. Instead, A_2_ and B_2_ supercell simulations do not show this evolution because the chain position in the on-surface lattice already allows them to find space vertically. Despite none of the four bulk simulations evolved to a structure close to that of the bulk polymorph observed experimentally—a result which could simply mean that for DCV-3 the organization on flat surfaces is very different from that in the bulk—the predicted structures share some features of the bulk cell. In fact, B structures present the same experimental intralayer organization, while a different planar disposition is observed for A classes, which however have larger cohesion energies. This specific planar arrangement affects the propyl chain interlayer insertion (Figure 6), which in A_1_ cell does not happen in cavities between two dicyanovinyl units but between sulfur atoms of vicinal thiophene and bithiophene units. Instead, the larger *xy* shift found between each layer of A_2_ morphology allows for the proximity of alkyl chains with cyano groups in the upper layer, similarly to the experimental cell. B_1_ structure resembles the most the experimental bulk one but a slightly larger *xy* shift causes an incomplete chain insertion and a less packed morphology. Interestingly, in B_2_ an even larger shift causes the insertion of chains of opposite faces within the same cavity. Compared to DCV-1 and DCV-2, the longer alkyl chains of DCV-3 increases the number of possible contacts between nitrogen atoms and aliphatic hydrogens (Figure S10), making this type of hydrogen bond formation more likely to occur than that with aromatic hydrogens.

**Figure 6:**
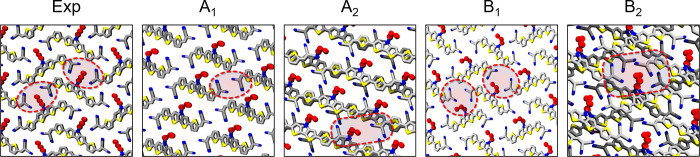
Focus on the position of the propyl chain (red, balls and sticks) with respect to the cavity formed by two neighboring dicyanovinyl groups (shaded areas surrounded by dashed red lines) for all the DCV-3 polymorphs. Molecules of A_2_ and B_2_ structures are colored with different shades for better visualization of the interlayer shift.

## Conclusions

This study introduces a novel scheme for predicting the formation of organic crystalline structures on surfaces, addressing a critical step in the fabrication of thin-film systems. The developed method allows for a layer-sequential investigation of stable crystalline arrangements and the identification of surface-induced polymorphs (SIPs). Furthermore, it enables the exploration of possible structural transitions between on-surface and bulk phases, offering a comprehensive framework for analyzing molecular organization, which often differs at surface-level from that observed in thick films or single crystals. The method provided a good match with experimental crystalline parameters and arrangements of a set of six test organic semiconductors, including acenes and dicyanovinyl molecules.

Currently, MYTHOS is limited to the morphological investigation of single-conformer crystals and effectively predicts only crystalline structures of molecules in a recumbent position on the surface, unless the (user-defined) size of the initial aggregates is large enough to stabilize the vertical arrangement. As scrutinized in Table 7, alongside possible future improvements, several other limitations stemmed from the approximations required to keep MYTHOS computationally affordable.

Nevertheless, this fully classical SCSP method offers key advantages in simulating real on-surface crystals. It incorporates surface and kinetic effects that influence crystal growth while also accounting for possible disorder and defects, enabling comparisons between crystalline structures formed on surfaces and those created in bulk environments. Taking that into account, the method is intended as a tool for the initial screening of realistic structures to provide a starting geometry for refinement at the QM level with, for instance, periodic DFT calculations.

Since interfacial regions play a crucial role in determining the electronic properties of organic electronic devices, such as organic solar cells, understanding these surface-level organizations is essential to design surface-tailored organic materials and enhance device performance and functionality.

**Table 7:**
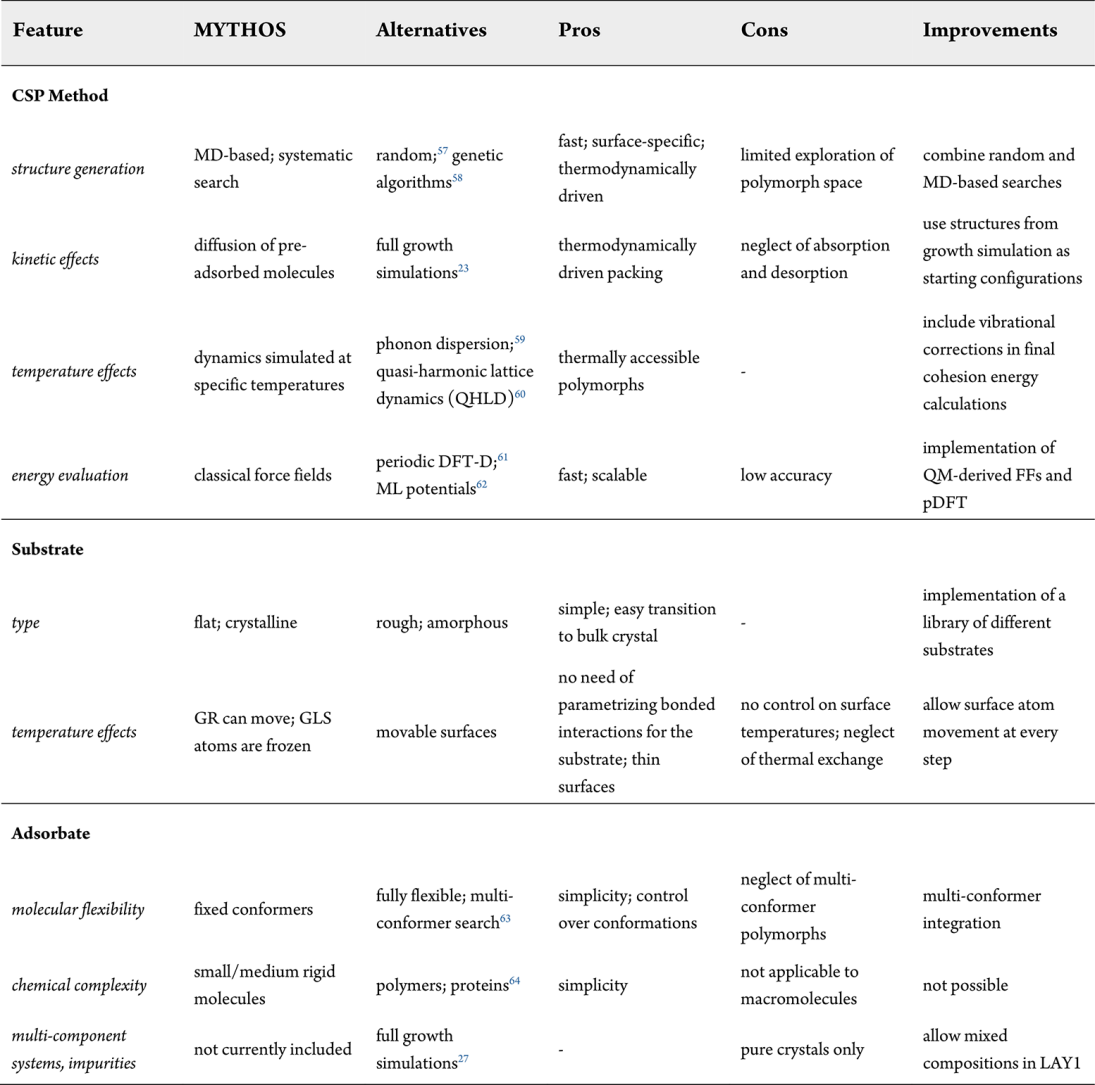
Comparison of MYTHOS and Alternative CSP Schemes, Highlighting Strengths and Limitations and Possible Improvements of the MYTHOS Approach^57,58,59,60,61,62,63,64^

## Supplementary Material





## Data Availability

MYTHOS is written in Python3 and takes advantage of some external utilities intrinsic to Unix and Unix-like operating systems. The program is interfaced to the freely distributed computational software NAMD^49^ for molecular dynamics calculations and VMD for molecular visualization. The code is available for download from GitLab at the following link: https://gitlab.com/emilio.lorini/MYTHOS. Detailed information concerning the technical aspects of the method is given in the manual therein.

## Notes

MYTHOS workflow for each molecule (PDF)
Parameters and topology files, and crystalline cell data (ZIP)

## Notes

The following CSD entries contain the supplementary crystallographic data for each molecule treated in this paper. PEN: 1230799, 619981; PFP: 234729; α-6T: 1309240, 1309241; DCV-1: 850004; DCV-2: 2392619; DCV-3: 2392620. These data can be obtained free of charge from The Cambridge Crystallographic Data Centre via www.ccdc.cam.ac.uk/data_request/cif.
