# Supplementary material for: MYTHOS: A Python Interface for Surface Crystal Structure Prediction of Organic Semiconductors
Source: J Chem Inf Model. 2025 Jul 9;65(14):7619–31. doi: 10.1021/acs.jcim.5c00669 (PMC12308790; doi:10.1021/acs.jcim.5c00669)
Supplement: Supplementary file 1 [file ci5c00669_si_001.pdf]

Supporting Information

MYTHOS: A Python Interface for Surface Crystal Structure Prediction of Organic Semiconductors

Emilio Lorini*,[1] Karsten Walzer,[2] Martin Pfeiffer,[2] Luca Muccioli[1]

[1] Department of Industrial Chemistry, University of Bologna, Via Piero Gobetti, 85, 40129, Bologna, Italy.
[2] Heliatek GmbH, Treidlerstraße 3, 01139, Dresden, Germany.

Index

Test molecules **2**

Calculation of adsorption energy **3**

Workflow for each molecule **4**

Time scale 4

PEN 5

PFP 8

α-6T 11

DCV-1 17

DCV-2 21

DCV-3 26

Hydrogen bonds **30**

References **36**

Test molecules

Abbreviation	Technical name	SMILES code	IUPAC name
PEN	pentacene	c1c2cc3cc4ccccc4cc3cc2cc2ccccc12	2,3:6,7-Dibenzanthracene
PFP	perfluoropentacene	Fc1c2c(c(F)c3c(F)c4c(F)c5c(c(F)c4c(F)c3c2F)c(F)c(F)c(F)c5F)c(F)c(F)c1F	1,2,3,4,5,6,7,8,9,10,11,12,13,14-Tetradecafluoropentacene
α-6T	α-sexithiophene	c1cc(sc1c1ccc(s1)c1cccs1)c1ccc(s1)c1ccc(s1)c1cccs1	2,2′:5′,2″:5″,2‴:5‴,2′‴:5⁗,2⁗′-Sexithiophene
DCV-1	DCV5T-Me_2	N#CC(=Cc1ccc(s1)c1ccc(s1)c1sc(c(c1C)C)c1ccc(s1)c1ccc(s1)C=C(C#N)C#N)C#N	2,2′-[(3′′,4′′-Dimethyl[2,2′:5′,2′′:5′′,2′′′:5′′′,2′′′′-quinquethiophene]-5,5′′′′-diyl)dimethylidyne]bis[propanedinitrile]
DCV-2	DCV-Fu-PyT-Fu-iPr	N#C/C(=C\c1ccc(o1)c1cc2c(s1)cc(n2C(C)C)c1ccc(o1)/C=C(\C#N)/C#N)/C#N	2,2′-(((4-isopropyl-4H-thieno[3,2-b]pyrrole-2,5-diyl)bis(furan-5,2-diyl))bis-(methaneylylidene))dimalononitrile
DCV-3	DCV-T-TPyT-T-Pr	CCCn1c2cc(sc2c2c1cc(s2)c1ccc(s1)C=C(C#N)C#N)c1ccc(s1)C=C(C#N)C#N	2,2′-[(4-Propyl-4*H*-dithieno[3,2-*b*:2′,3′-*d*]pyrrole-2,6-diyl)bis(5,2-thiophenediylmethylidyne)]bis[propanedinitrile]

Calculation of adsorption energy

In order to adjust the Lennard-Jones (LJ) parameters of the GLS surface to resemble those of HOPG, we performed calculations of adsorption energy profiles for a chosen molecule, pentacene, on both surfaces. We employed the Adaptive Biasing Force (ABF) method[1] applied for a specific collective variable (colvar): the orthogonal distance from the surface. One pentacene molecule was let free to explore the space along that direction. To delimit the collective variable space, repulsive potential energy barriers were set at the borders of the colvar region, extending between 2 Å and 20 Å from the highest layer. By varying the depth of the LJ potential well of GLS (ε), good agreement of the two adsorption profiles was reached (Figure S1) with a $\varepsilon_{GLS} = 0.016$ kcal mol^{-1} with respect to $\varepsilon_{GR} = 0.086$ kcal mol^{-1}.

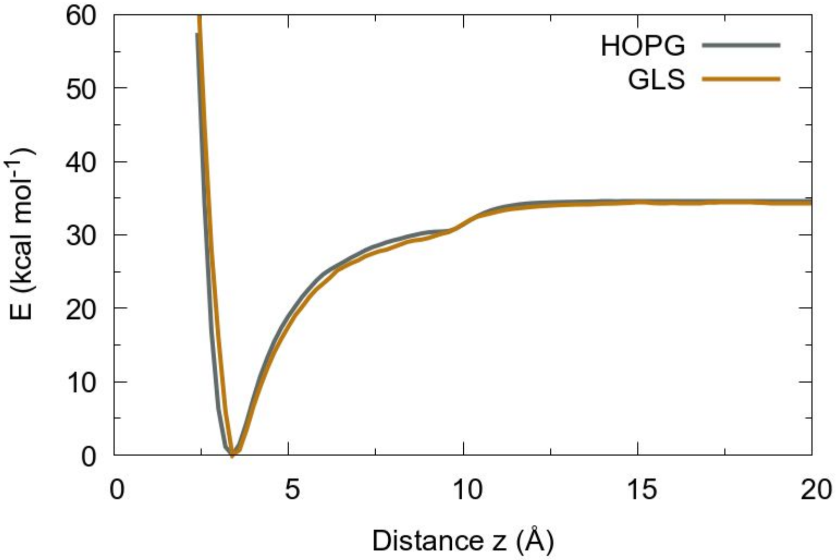

Figure S1. Adsorption free energy profile obtained moving vertically one molecule of PEN on the HOPG and GLS surface.

Workflow for each molecule

Time scale

Molecule	Atoms	LAY1			LAY2			BULK (s.p.)	
		MD	En	Min	MD	En	Min	FF	pDFT
PEN	36	219	5	19	295	12	32	-	-
PFP	36	227	6	20	297	12	35	-	-
α-6T	44	214	6	21	308	13	45	-	-
DCV-1	55	236	7	22	335	15	49	-	-
DCV-2	48	229	6	19	314	13	44	-	-
DCV-3	51	228	7	21	324	15	46	0.21	272

Table S1. Approximate elapsed time (in minutes using 6 cores) required for generating the configuration ensemble through 10 ns molecular dynamics (MD) simulations of six molecules, for the calculation of the interaction energies in each frame (En) and for the energy minimization (Min) of the pre-minima ensemble (20 structures, 20 ps each). Data are shown for both the first (LAY1) and second (LAY2) layer investigations, and for all tested molecules. For reference, the approximate time required for a single-point energy calculation of a DCV-3 crystal cell is also provided at both the MD level on a supercell and periodic DFT (PBEsol functional) level. All simulations were performed on an Intel(R) Xeon(R) CPU E5-2620 v3 @ 2.40GHz.

PEN

**SET**	D_{2h} symmetry, no need for DF calculations
**LAY1**	Sequence of positional scans for planar aggregates of increasing size (2, 4 and 8 molecules). Planar unit cell (a = 16.449, b = 7.284, γ = 103.4) was extracted from the most stable organization (Figure S2a).
**BUILD1**	The planar unit cell was replicated 6 × 12 times to build a full monolayer of 72 molecules with an area of 98.692 × 87.412 Å^2. Such replica was placed on the GLS substrate and minimized.
**LAY2**	Positional scan of a 3 × 3 planar aggregate in the second layer indicated the presence of different tilt angle between molecules in the first and second layer. Planar cell containing one molecule for each layer (Z=2) was extracted from the MSMC (Figure S2b).
**BUILD2**	As done in BUILD1 step, the new cell was replicated 6 × 12 × 1 times and a full bilayer of 144 molecules was placed on the GLS substrate and minimized.
**LAY3**	Positional scan of a 3 × 3 planar aggregate in the third layer showed a most stable configuration of herringbone type with the same orientation between the first and third layer molecules (Figure S2c), from which the spatial cell was extracted.
**MULTI**	• The spatial cell was replicated 6 × 12 × 2 times to produce a four layers template, which was placed on the GLS substrate. Two types of simulations were performed: an energy minimization and MD simulations. The on-surface unit cell was extracted from the equilibration at 100 K after 1 ns (Figure S2d).<br>• The on-surface crystalline cell was replicated 6 × 6 × 4 times to build a supercell of 288 molecules (Figure S2e) that underwent a NVT step of 0.2 ns and an NPT one for 2 ns at 100 K. The bulk unit cell was extracted upon minimization of the system.<br><br>The on-surface and bulk lattice parameters are reported in Table 1.

a)

LAY1

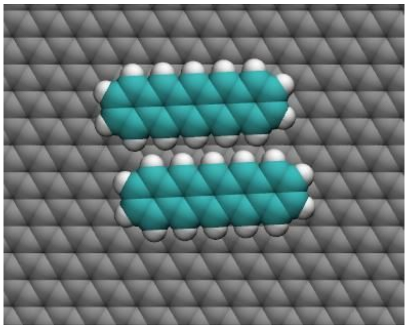

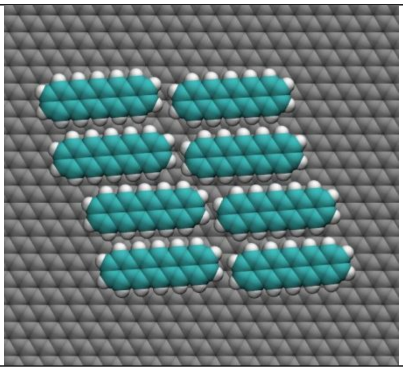

b)

LAY2

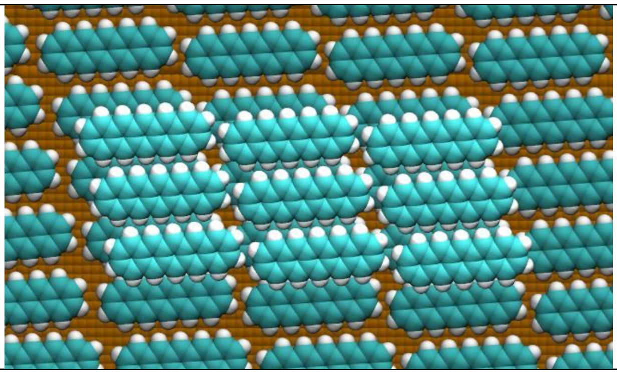

c)

LAY3

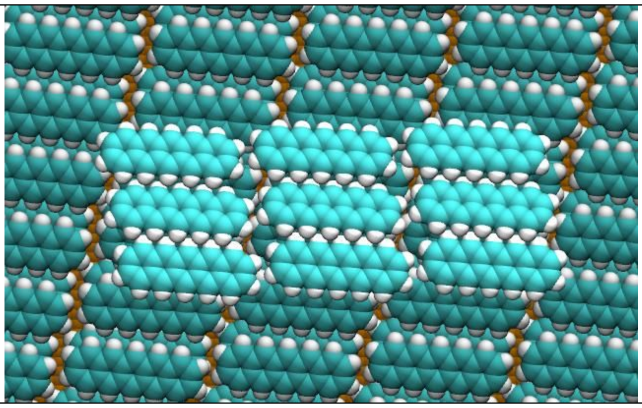

d)

MULTI

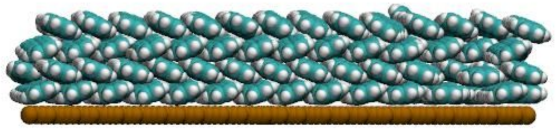

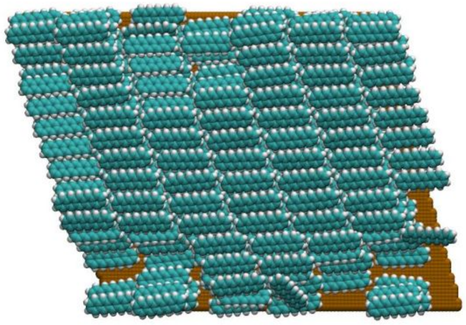

e)

BULK

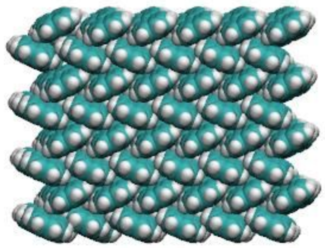

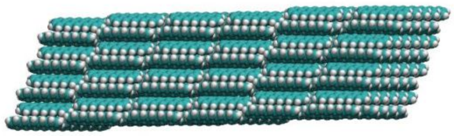

Figure S2. Different frames obtained from each MYTHOS stage for pentacene molecule: a-c) MSMC upon scanning of each investigated layer, d) four layers upon 100 K dynamics, e) minimized crystal in bulk environment after equilibration at 300 K.

PFP

**SET**	D_{2h} symmetry, no need for DF calculations
**LAY1**	Sequence of positional scans for planar aggregates of increasing size (2 and 4 molecules). Planar unit cell (a = 17.498, b = 8.578, γ = 75.4) was extracted from the most stable organization (Figure S3a).
**BUILD1**	The planar unit cell was replicated 6 × 12 times to build a full monolayer of 72 molecules with an area of 104.985 × 102.935 Å^2. Such replica was placed on the GLS substrate and minimized.
**LAY2**	Positional scan of a 2 × 2 planar aggregate in the second layer produced one MSMC (Figure S3b) with flat-lying configuration from which a spatial cell with Z=1 was extracted.
**MULTI**	• The spatial cell was replicated 6 × 12 × 4 times to produce a four layers template, which was placed on the GLS substrate. Two types of simulations were performed: energy minimization and MD simulations. The on-surface unit cell was extracted from the equilibration at 300 K after 1 ns (Figure S2c).<br><br>• The on-surface crystalline cell was replicated 5 × 8 × 8 times to obtain a supercell of 320 molecules that underwent a NVT step of 0.2 ns and an NPT one for 5 ns at different temperatures (300 K, 500 K, 700 K). The bulk unit cell was extracted from the system equilibrated at 300 K (Figure S2d) upon minimization.<br><br>The on-surface and bulk lattice parameters are reported in Table 2.

a)

LAY1

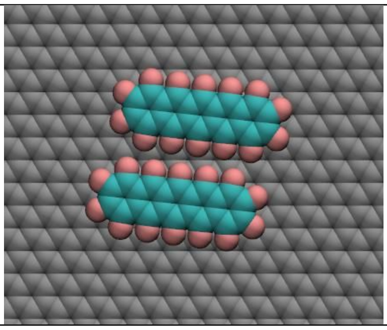

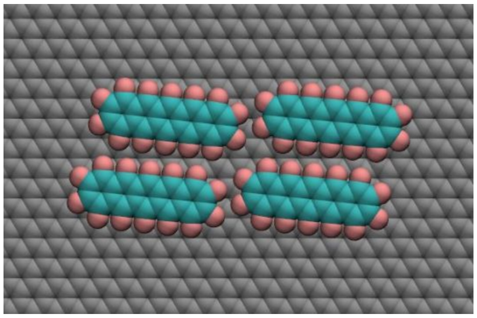

b)

LAY2

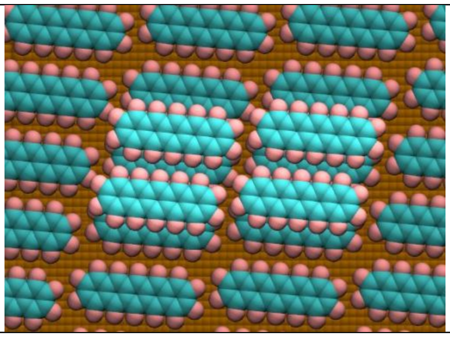

d)

MULTI

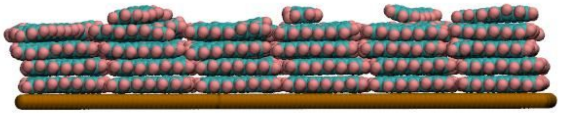

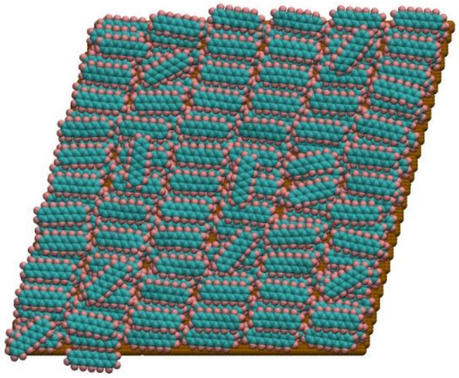

e)

BULK

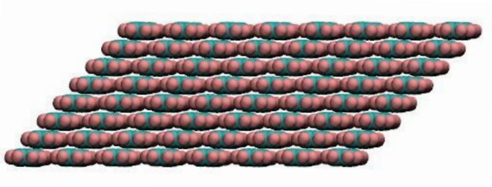	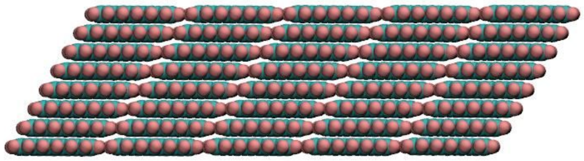

Figure S3. Different frames obtained from each MYTHOS stage for perfluoropentacene molecule: a-b) MSMC upon scanning of each investigated layer, c) four layers upon 300 K dynamics, e) minimized crystal in bulk environment after equilibration at 300 K.

α-6T

<table>
<tr><td>SET</td><td colspan="2">Conformer found in experimental polymorphs[2,3] (Figure 1) with C_{2h} symmetry: SF and DF calculations need to be performed.</td></tr>
<tr><td>LAY1</td><td colspan="2">SF
• MD simulation of 4 molecules at 300 K produced a stable side-side tetramer.
• One subsequent positional scan of two tetramers was performed for the edge-edge interaction and the planar unit cell (a = 26.168, b = 6.459, γ = 86.2) was extracted from the MSMC (Figure S4a), called class A.
DF
• A stable side-side dimer was obtained from a MD simulation of 4 molecules at 300 K (Figure S4a).
• Subsequent positional scan of two dimers and two tetramers were performed and a planar unit cell (a = 30.341, b = 15.227, γ = 49.7) with Z=2 was extracted from the most stable 8 molecule aggregate (Figure S4a), called class B.</td></tr>
<tr><td>BUILD1</td><td>Class A
A full monolayer with area of 96.888 × 130.841 $Å^2$ was built for class A by replicating 15 × 5 times the planar unit cell and minimized on top of the GLS substrate.</td><td>Class B
A full monolayer with area of 121.813 × 151.704 $Å^2$ was built for class B by replicating 8 × 5 times the planar unit cell and minimized on top of the GLS substrate.</td></tr>
<tr><td>LAY2</td><td>Two types of positional scan of a 3 × 2 planar aggregate in the second layer: one with molecules showing the same face of the first layer (SF), and one the opposite face (DF). A spatial cell was extracted from the most stable SF structure (class $\mathbf{A_1}$) while a new planar cell with Z=1 in each layer was extracted from the most stable DF structure (class $\mathbf{A_2}$).</td><td>A positional scan of a 8 molecules planar aggregate in the second layer was performed. A spatial cell was extracted from the most stable structure.</td></tr>
</table>

**BUILD2**	**Class A_2**<br>The $\mathbf{A_2}$ unit cell was replicated 15 × 5 times to build a bilayer template which was minimized on the GLS substrate.
**LAY3**	Positional scan of a 3 × 2 planar aggregate at the third layer. The complete spatial cell of class $\mathbf{A_2}$ with both faces (Z=2) was then extracted from the most stable morphology at the third layer.
**MULTI**	• Four full layers were built and the final on-surface crystalline cell was extracted for $\mathbf{A_1}$, $\mathbf{A_2}$ and **B** classes upon minimization. For the latter, the presence of a tilt angle within the latter imposed the extraction of a Z=4 unit cell.<br>• We replicated each on-surface unit cell 8 × 5 × 3 times for $\mathbf{A_1}$ and $\mathbf{A_2}$ configurations to build a supercell (240 molecules). Instead, B unit cell was replicated 4 × 4 × 5 times to build a supercell of 320 molecules. Bulk simulations were performed at 300 K for A_1 and B, while simulation at 150 K for A_2 produced a lower energy.<br>The on-surface and bulk lattice parameters for each class are reported in Table 3.

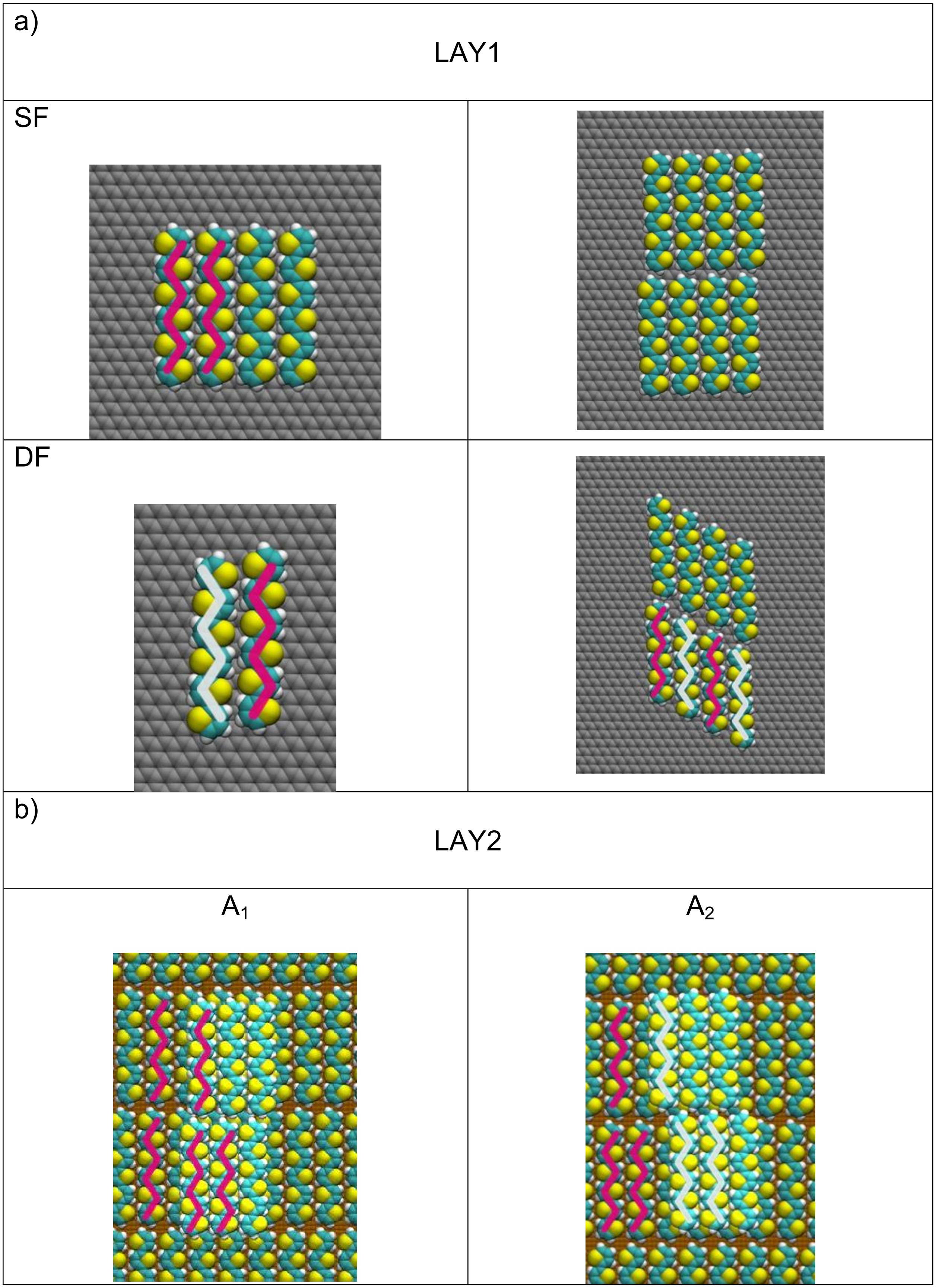
a)
LAY1
SF
DF
b)
LAY2
A1
A2

B

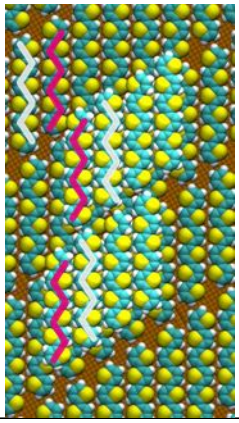

c)

LAY3

A_2

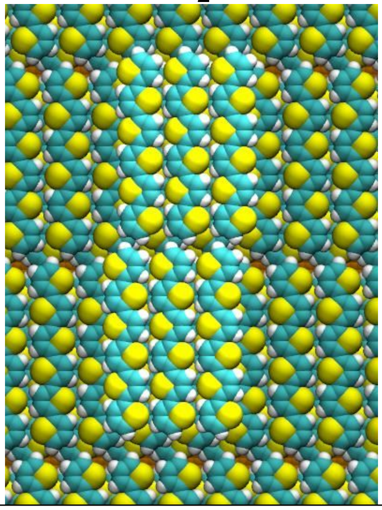

d)

MULTI

A_1

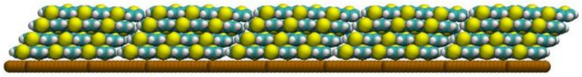

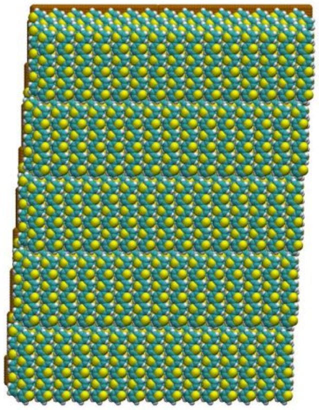

A_2

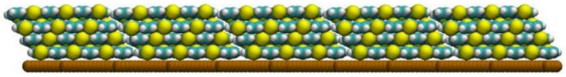

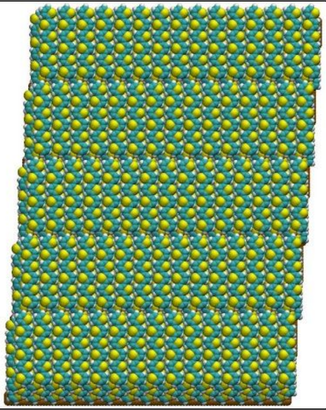

B

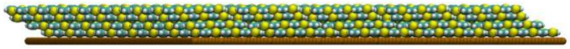

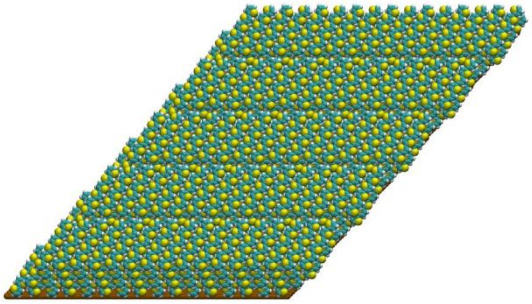

e)

BULK

A$_1$

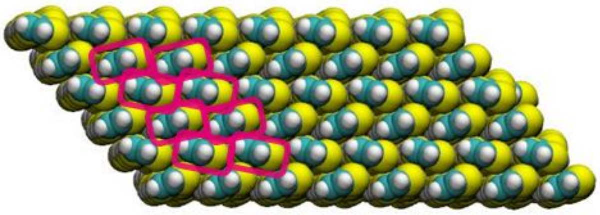

A$_2$

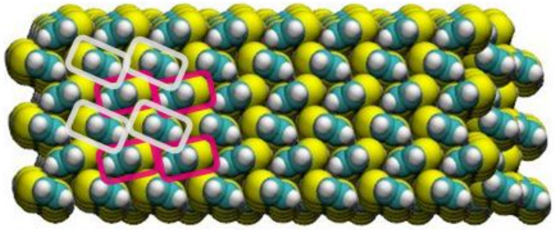

B

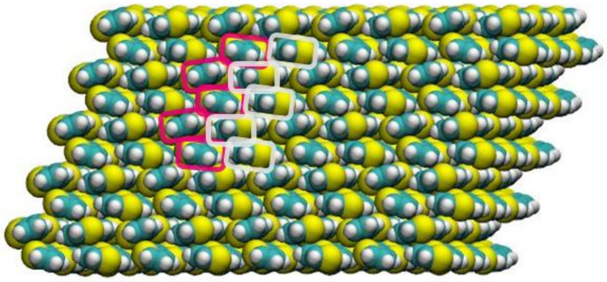

Figure S4. Different frames obtained from each MYTHOS stage for α-sexitophene: a-c) MSMC upon scanning of each investigated layer, d) four layers upon 300 K dynamics, e) minimized crystal in bulk environment after equilibration at 300 K.

DCV-1

**SET**	The conformer found in the crystalline cell obtained from XRD[4] (Figure 1) has C_{2v} symmetry and, therefore, DF calculations were not needed. Molecular interconversion was possible at high temperatures, therefore, four torsion angles highlighted in the figure were blocked within the force field over all simulations.
**LAY1**	• MD simulations with different number of molecules diffusing at 700 K produced many stable aggregates. The planar unit cell of class **A** (a = 18.103, b = 27.339, γ = 109.14) with Z=2 was extracted from a stable pentamer (Figure S5a).<br><br>• Sequential positional scans revealed the presence of a second stable 2D configuration (class **B**) which lattice parameters (a =17.721, b = 52.315, γ = 93.35, Z=4) were extracted from a 9 molecules aggregate.
**BUILD1**	**Class A**<br>A full monolayer with area of 108.618 × 136.695 Å^2 was built for class **A** by replicating 6 × 5 times the planar unit cell and minimized on top of the GLS substrate.<br><br>**Class B**<br>A full monolayer with area of 88.562 × 156.962 Å^2 was built for class **B** by replicating 5 × 3 times the planar unit cell and minimized on top of the GLS substrate.
**LAY2**	A positional scan of a planar aggregate (3 × 2 for **A** and 2 × 2 for **B**) in the second layer was performed on top of the minimized systems. The 3D cell was extracted from the two most stable structures of each class, which were differing for the positional shift of the LAY2 aggregate respect to the first layer (Figure S5b): $\mathbf{A_1}$, $\mathbf{A_2}$, $\mathbf{B_1}$ and $\mathbf{B_2}$.
**MULTI**	• Six full layers were built and equilibrated for 1 ns at 500 K on the GLS substrate for each class. After minimization, we observed the formation of a tilt angle respect to the surface plane. The final on-surface crystalline cells were extracted from the molecules in the third layer and are reported in Table 4.<br><br>• We replicated the on-surface unit cell of each class 3 × 4 × 6 times to build a supercell (288 molecules) and perform a bulk simulation at 500 K with a single NPT step of 2.0 ns. The bulk crystalline parameters for each class are reported in Table 4.

a)

LAY1

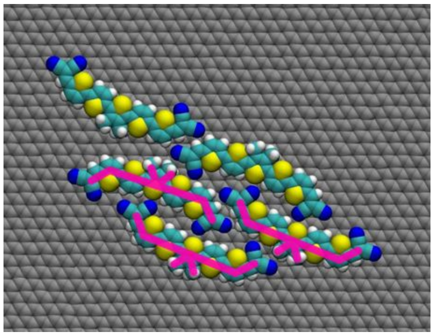

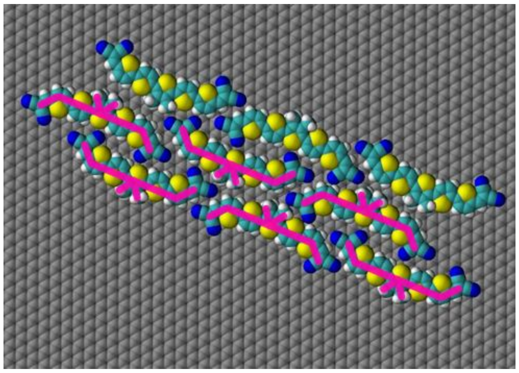

b)

LAY2

A_1

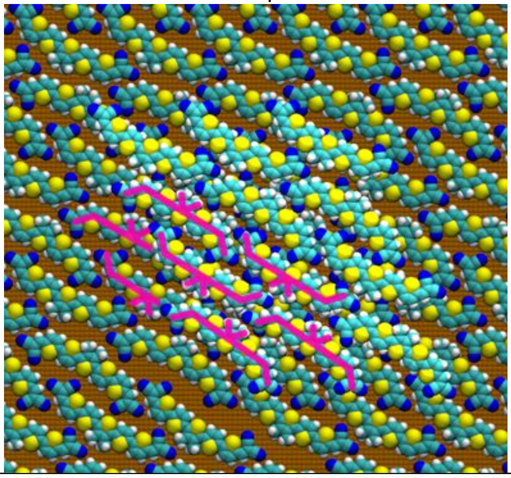

A_2

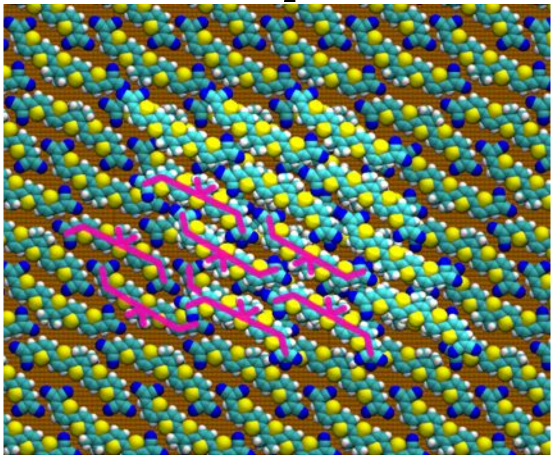

B_2

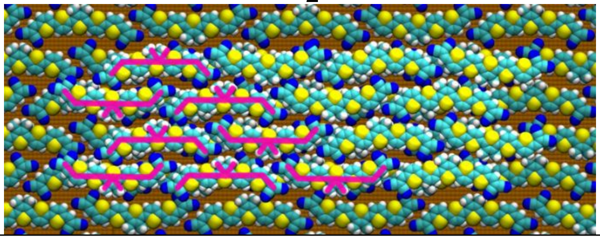

c)

MULTI

A_1

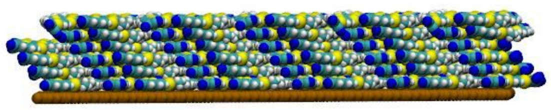

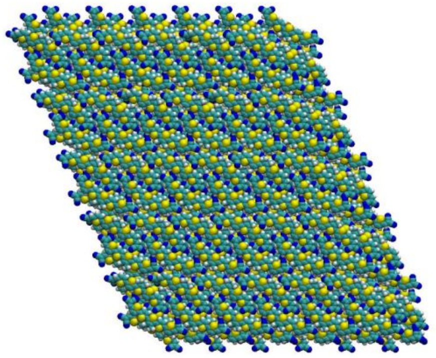

A_2

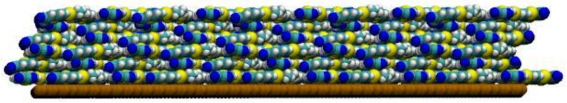

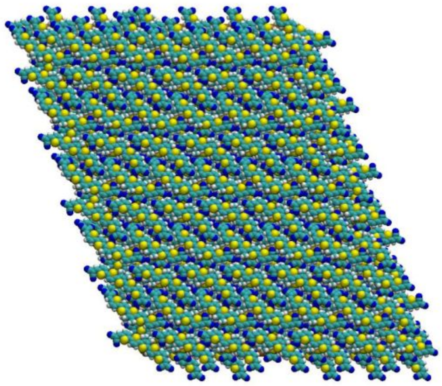

B_1

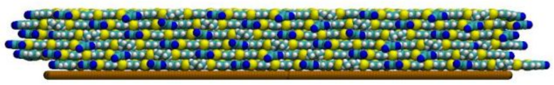

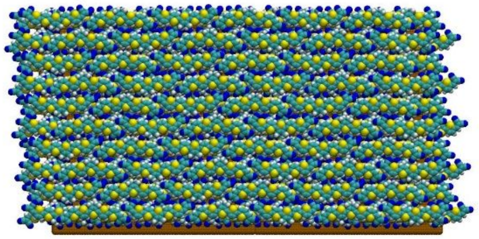

B_2

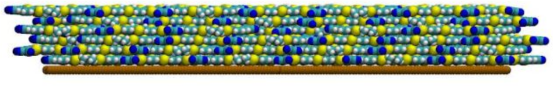

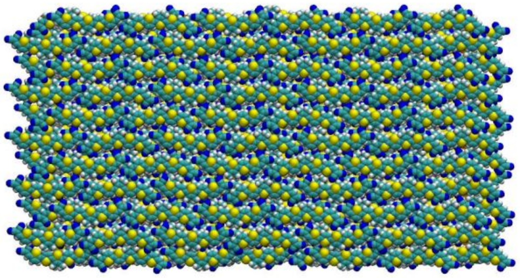

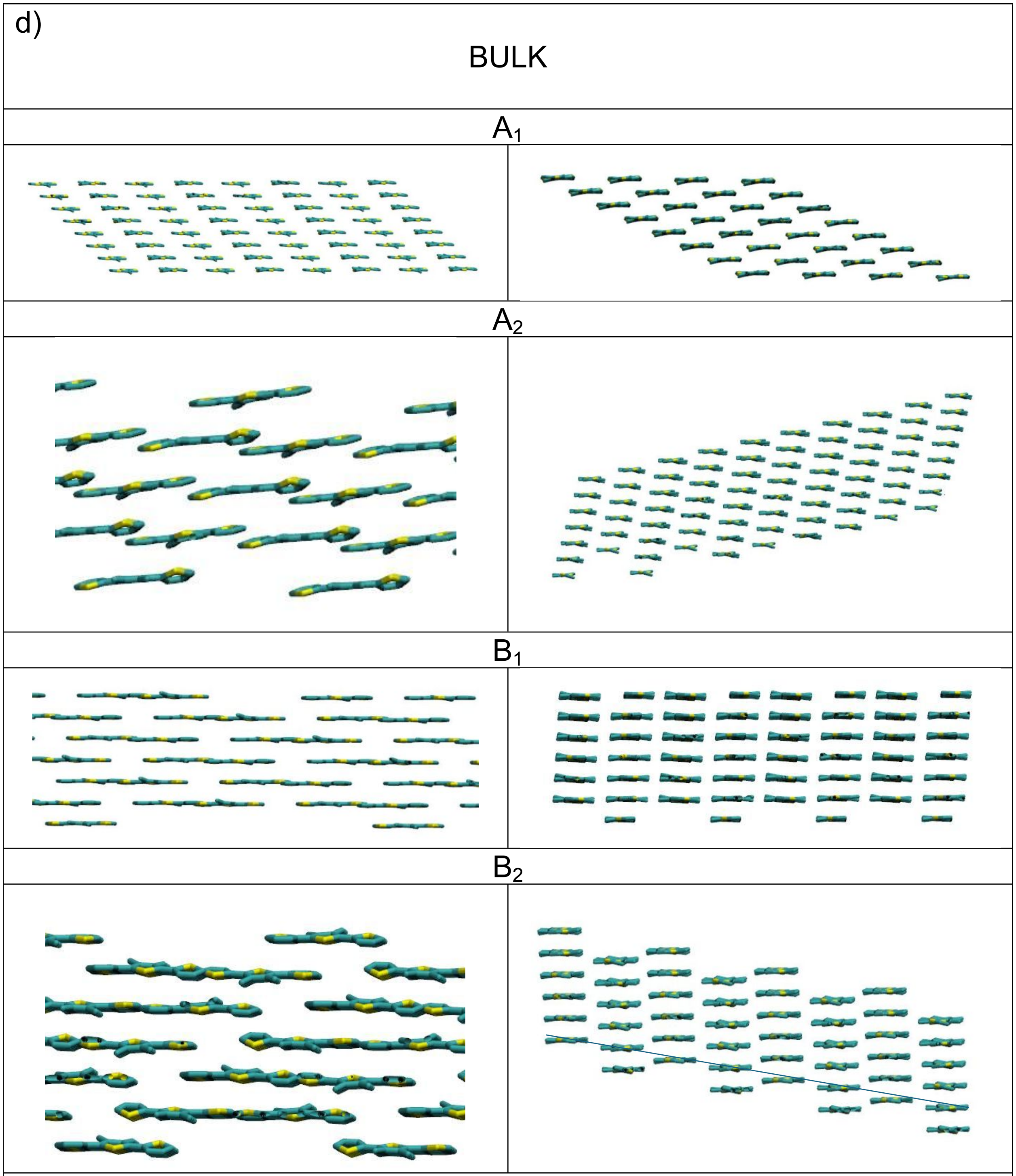

Figure S5. Different frames obtained from each MYTHOS stage for DCV-1: a-b) MSMC upon scanning of each investigated layer, d) six layers upon 500 K dynamics, e) molecular cores of minimized crystal in bulk environment after equilibration at 500 K.

DCV-2

**SET**	Conformer found in the crystalline cell measured with XRD[5] (Figure 1) with C_s symmetry: SF and DF calculations need to be performed. Molecular interconversion was possible at high temperatures, therefore, four torsion angles highlighted in the figure were blocked within the force field over all simulations.		
**LAY1**	• MD simulations at 700 K produced stable aggregates in SF calculations (Figure S6a). The planar unit cell was extracted for both classes **A** (a = 8.852, b = 34.296, γ = 95.67) with Z=2 and **B** (a = 8.839, b = 17.148, γ = 83.73) with Z=1.<br>Instead, only stable dimers were detected from DF simulations.<br><br>• Positional scan of the most stable DF dimer and a subsequent scan of the most stable DF tetramer allowed the formation of a 8 molecule aggregate (Figure S6a), which permitted the extraction of the planar unit cell (a = 9.017, b = 35.707, γ = 102.8) of class **C** containing one molecule for each face (Z=2).		
	**Class A**	**Class B**	**Class C**
**BUILD1**	A full monolayer with area of 88.520 × 102.888 Å^2 was built for class **A** by replicating 10 × 6 times the planar unit cell and minimized on top of the GLS substrate.	A full monolayer with area of 88.390 × 102.888 Å^2 was built for class **B** by replicating 10 × 6 times the planar unit cell and minimized on top of the GLS substrate.	A full monolayer with an area of 90.170 × 107.122 Å^2 was built for class **C** by replicating 10 × 3 times the planar unit cell and minimized on top of the GLS substrate.
**LAY2**	Positional scan of a 3 × 2 planar aggregate of 12 molecules at the second layer. The 3D cell of class **A** (Z=2) was extracted from the most stable morphology at the second layer (Figure S6b).	Positional scan of a 4 × 3 planar aggregate of 12 molecules at the second layer. A new planar cell with Z=1 in each layer was extracted from the most stable structure.	Positional scan of a 3 × 2 planar aggregate of 12 molecules at the second layer. A new planar cell with Z=2 in each layer was extracted from the most stable structure.
**BUILD2**		A full bilayer with an area of 88.786 × 101.644 Å^2 was built for class **B** by replicating 10 × 6 times the new planar unit cell and minimized on top of the GLS substrate.	A full bilayer with an area of 90.028 × 103.765 Å^2 was built for class **C** by replicating 10 × 3 times the new planar unit cell and minimized on top of the GLS substrate.

**LAY3**		Positional scan of a 2 × 2 planar aggregate at the third layer. The 3D cell of class **B** with both faces (Z=2) was then extracted from the most stable morphology at the third layer (Figure S6c).	Positional scan of a 3 × 2 planar aggregate at the third layer. The 3D cell of class **C** with both faces (Z=4) was then extracted from the most stable morphology at the third layer.
**MULTI**	• Six full layers were built for **A**, **B** and **C** class and the final on-surface crystalline cell was extracted upon equilibration at 500 K for 1 ns and minimization (Figure S6d).<br>• We replicated 4 × 4 × 4 times the on-surface unit cells of **A** and **B** classes and 4 × 3 × 3 the unit cell of **C** class to build a supercell (128 and 144 molecules) and perform a bulk simulation at 150 K with a NVT step of 0.2 ns and NPT of 2.0 ns (Figure S6e).<br>The on-surface and bulk lattice parameters for each class are reported in Table 5.		

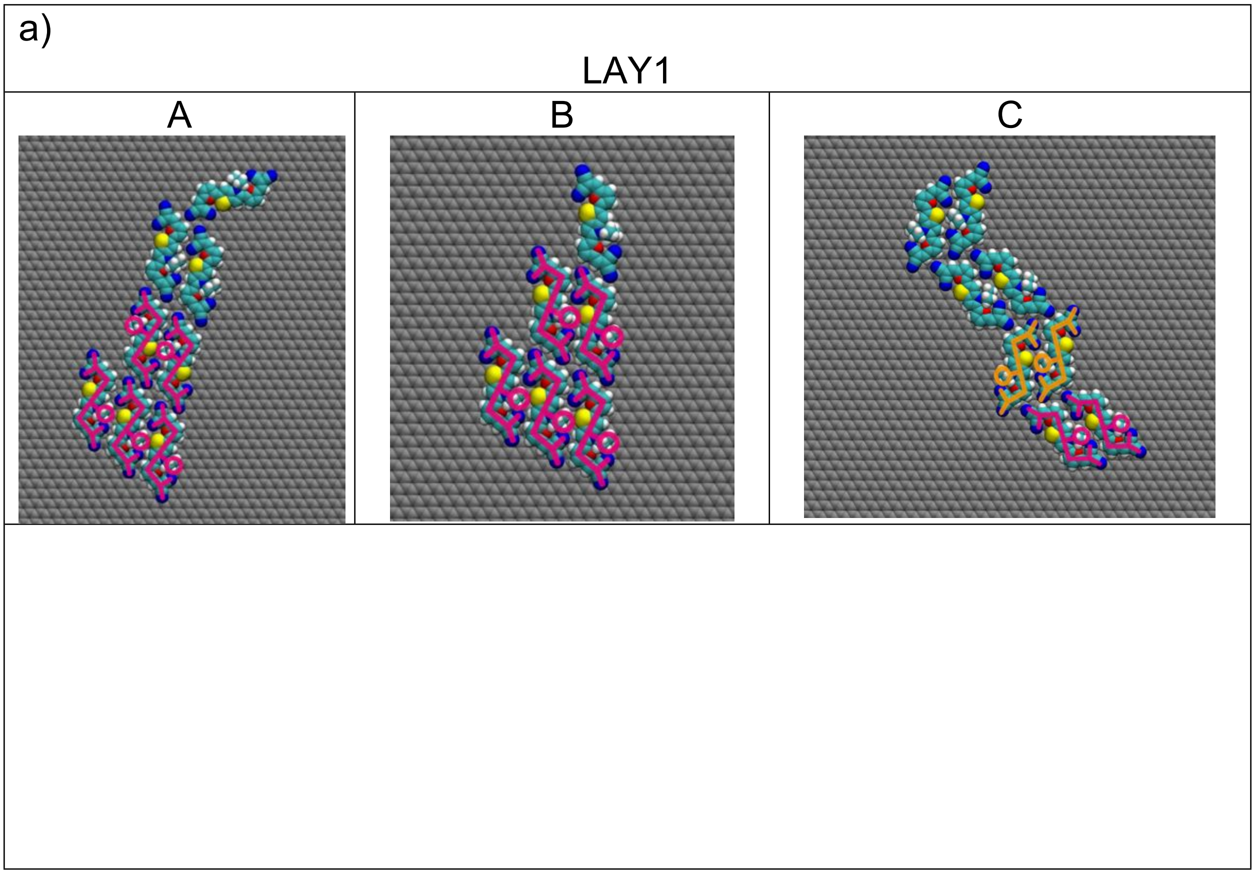

b)

LAY2

A

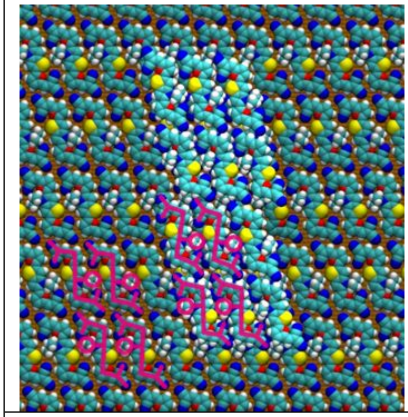

B

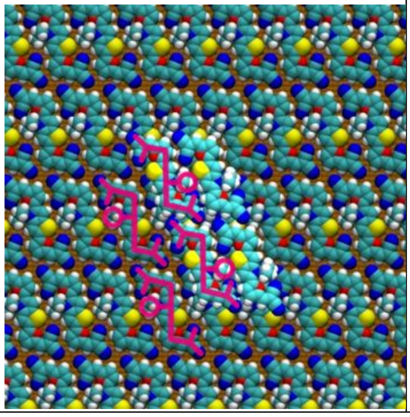

C

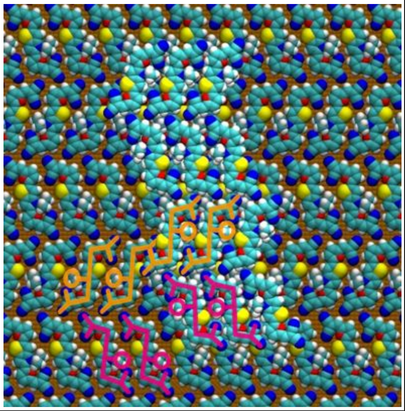

c)

LAY3

B

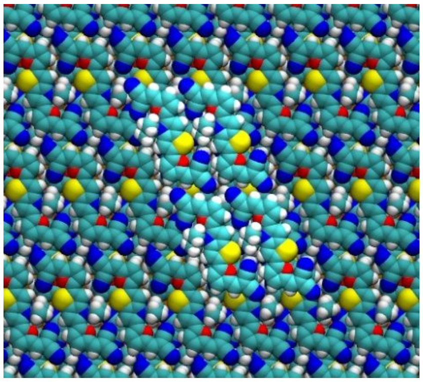

C

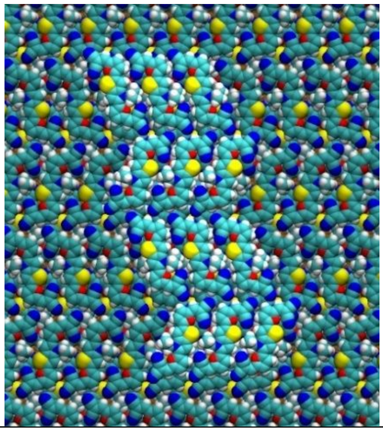

d)

MULTI

A

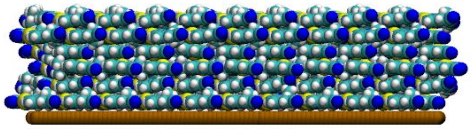

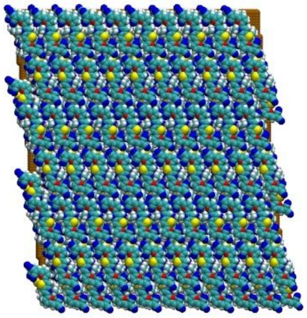

B

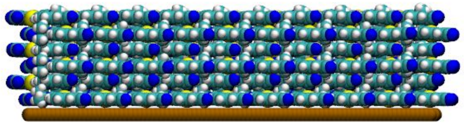

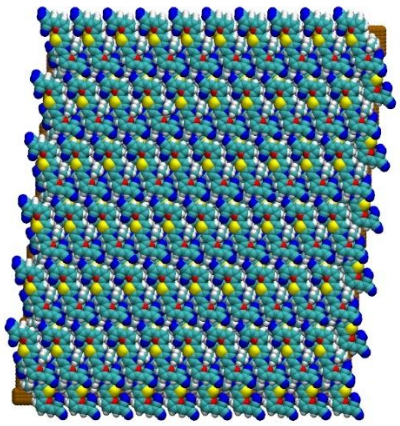

C

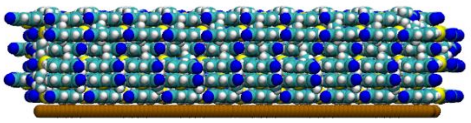

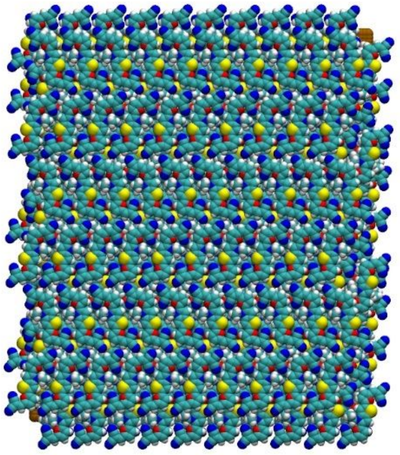

e)

BULK

A

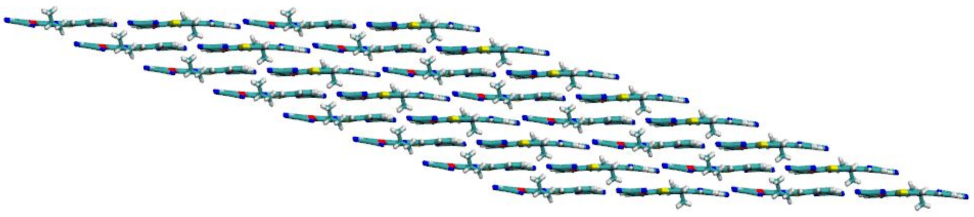

B

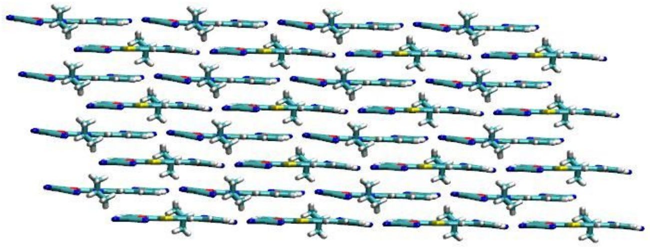

C

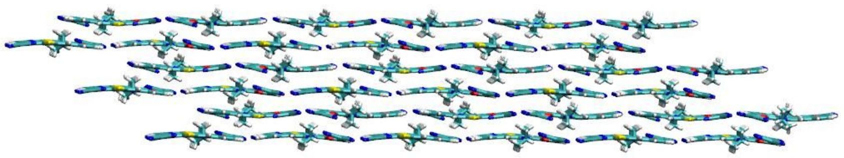

Figure S6. Different frames obtained from each MYTHOS stage for DCV-2: a-c) MSMC upon scanning of each investigated layer, d) four layers upon 500 K dynamics, e) minimized crystal in bulk environment after equilibration at 150 K.

Table S2. Experimental and predicted crystalline cells of DCV-2 molecules in bulk with reparametrized Dreiding and GAFF force fields: lattice sides (Å), angles (°), molecular units per cell (Z), volume per molecule (nm^3) and cohesion energy per molecule (kcal/mol).

		a	b	c	α	β	γ	Z	V	E^{COH}
Exp		8.7	17.5	6.8	90.0	90.0	90.8	2	0.520	-125.1
Dreiding	A	8.7	35.4	11.3	20.3	104.3	95.4	2	0.535	-120.0
	B	8.8	17.3	7.3	76.6	89.7	84.3	2	0.539	-121.9
	C	8.8	33.9	13.2	32.9	89.9	89.3	4	0.530	-125.0
GAFF	A	8.8	34.3	11.1	23.3	106.3	96.6	2	0.593	-123.9
	B	8.9	16.9	7.2	76.9	89.6	84.1	2	0.525	-128.9
	C	8.9	34.7	13.3	31.5	89.9	89.3	4	0.536	-127.0

DCV-3

**SET**	Conformer found in the crystalline cell measured with XRD[5] (Figure 1) with C_S symmetry: SF and DF calculations need to be performed. Molecular interconversion was possible at high temperatures, therefore, four torsion angles highlighted in the figure were blocked within the force field over all simulations.
**LAY1**	• MD simulations with high number of molecules diffusing at 700 K produced many disordered structures for both SF and DF calculations. A stable tetramer (Figure S7a) was obtained from a SF simulation of 4 molecules and the planar unit cell (a = 9.727, b =27.064, γ = 47.1) of class **A**, containing one molecular face, could be extracted. Instead, different types of dimers could be evaluated from a 2 molecules DF simulation.<br><br>• Positional scan of the most DF stable dimer and a subsequent scan of the most stable DF tetramer allowed the formation of a 8 molecule aggregate (Figure S7a), which permitted the extraction of the planar unit cell (a = 14.433, b = 30.720, γ = 59.7) of class **B** containing one molecule for each face (Z=2).
**BUILD1**	**Class A**<br>A full monolayer with area of 97.270 × 162.384 Å² was built for class A by replicating 10 × 6 times the planar unit cell and equilibrated at 700 K on top of the GLS substrate.<br><br>**Class B**<br>A full monolayer with area of 115.461 × 122.879 Å² was built for class A by replicating 8 × 4 times the planar unit cell and equilibrated at 700 K on top of the GLS substrate.
**LAY2**	• Scanning could not carry out accurate predictions for both classes due to the presence of the out-of-plane propyl side chain, which imposed a distance between the aggregate and the monolayer too large for a correct evaluation of the most stable interactions.<br><br>• For both **A** and **B** classes, MD calculations with a 2 × 2 constrained aggregate, with intermolecular harmonic potentials, in the second layer were performed at 700 K. From this approach, we could detect two main types of configurations for each class (Figure S7b) depending on the positional shift of the molecules in the second layer respect to bottom layer. The spatial unit cells were extracted from the configurations with minor shift ($\mathbf{A_1}$ and $\mathbf{B_1}$) and from the ones with larger shift ($\mathbf{A_2}$ and $\mathbf{B_2}$).
**MULTI**	• Six full layers were built and equilibrated at 300K on the GLS substrate for each class (Figure S7c). The final on-surface crystalline cells were extracted from the molecules in the third layer and reported in Table 6.

	• We replicated each on-surface unit cell 4 × 8 × 8 times for $\mathbf{A_1}$ and $\mathbf{A_2}$ configurations and 4 × 4 × 8 times $\mathbf{B_1}$ and $\mathbf{B_2}$ to build a supercell (256 molecules) and perform a bulk simulation at 300 K with a NVT step of 0.2 ns and NPT of 2.0 ns (Figure S7d). The final bulk unit cell are reported in Table 6.

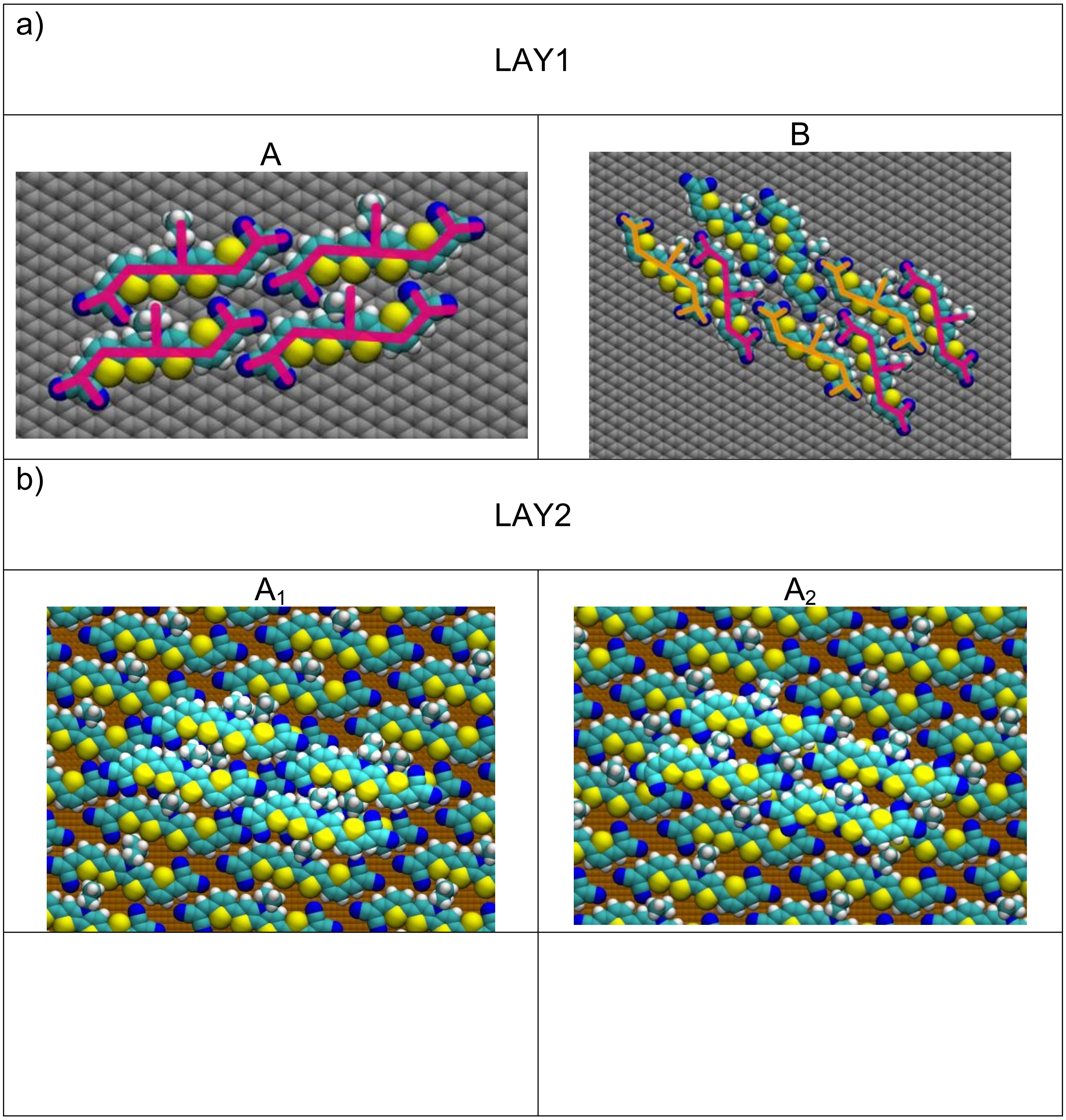

B1	B2
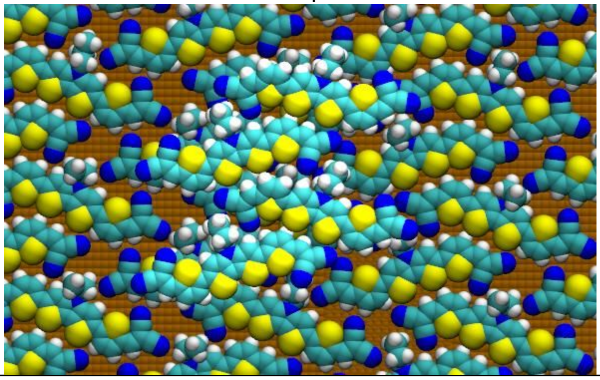	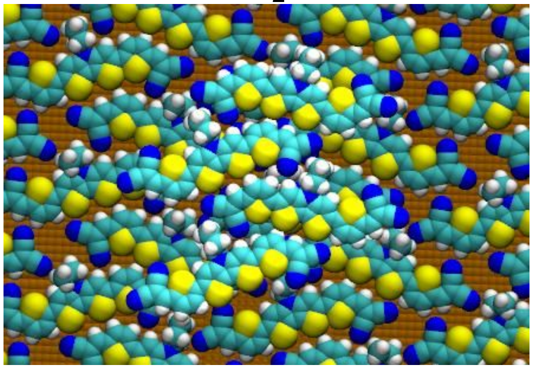

c)

MULTILAY

A1

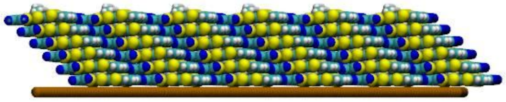	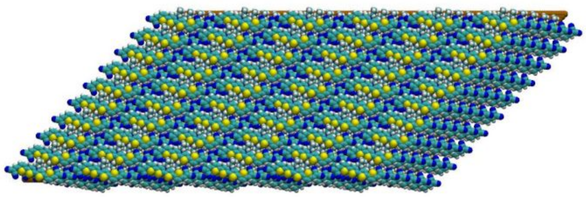

A2

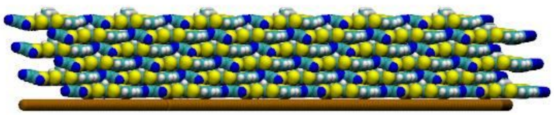	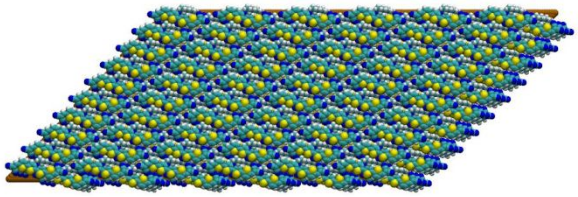

B1

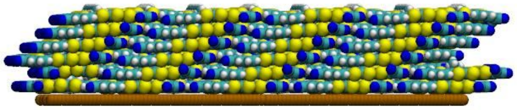	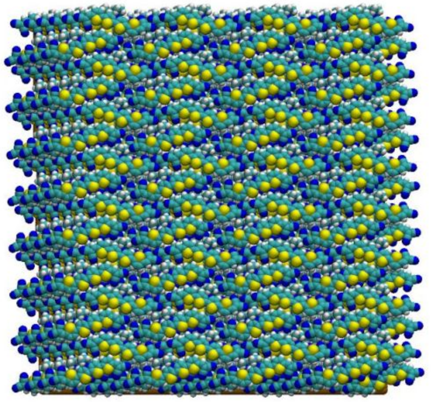

B$_2$

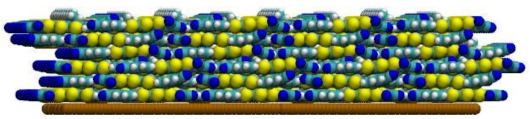

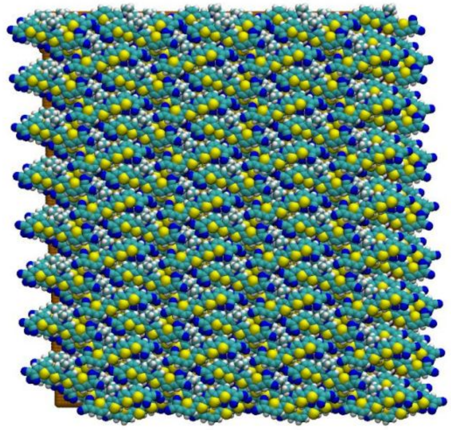

d)

BULK

A$_1$

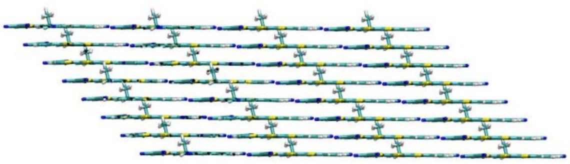

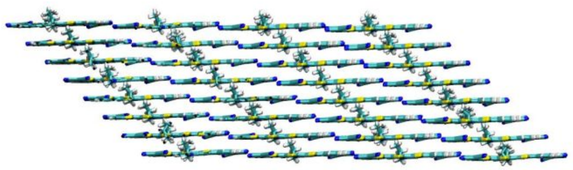

A$_2$

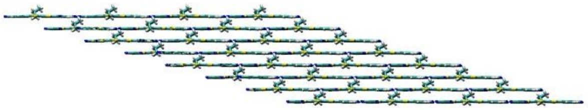

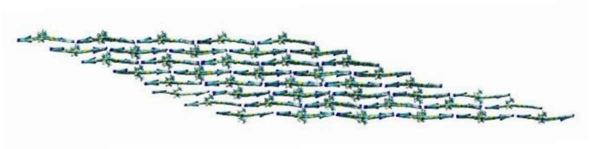

B$_1$

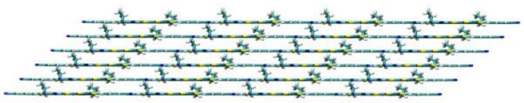

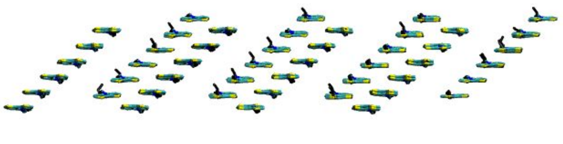

B$_2$

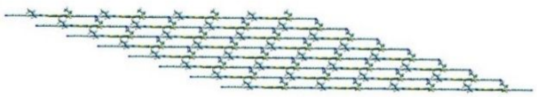

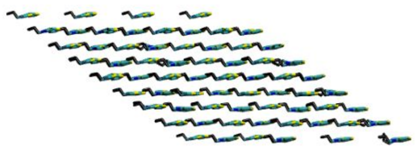

Figure S7. Different frames obtained from each MYTHOS stage for DCV-3: a-b) MSMC upon scanning of each investigated layer, d) four layers upon 300 K dynamics, e) molecular cores of minimized crystal in bulk environment after equilibration at 300 K.

Hydrogen bonds

The potential hydrogen bonding between molecules in the bulk crystalline structures of DCV-1, DCV-2 and DCV-3 was assessed by calculating the intermolecular distances between the nitrogen atoms of the dicyanovinyl groups and all hydrogen atoms, categorized as aliphatic (from alkyl chains) and aromatic (from conjugated core). The N--H distances shorter than 3 Å were averaged across all molecules, with only the smallest distance for each atom considered, recognizing that each atom can participate in only one bond. Additionally, the angles between the hydrogen bond vector and the corresponding C-H bond were calculated, and the resulting distribution is reported for each polymorph.

DCV-1

A_1

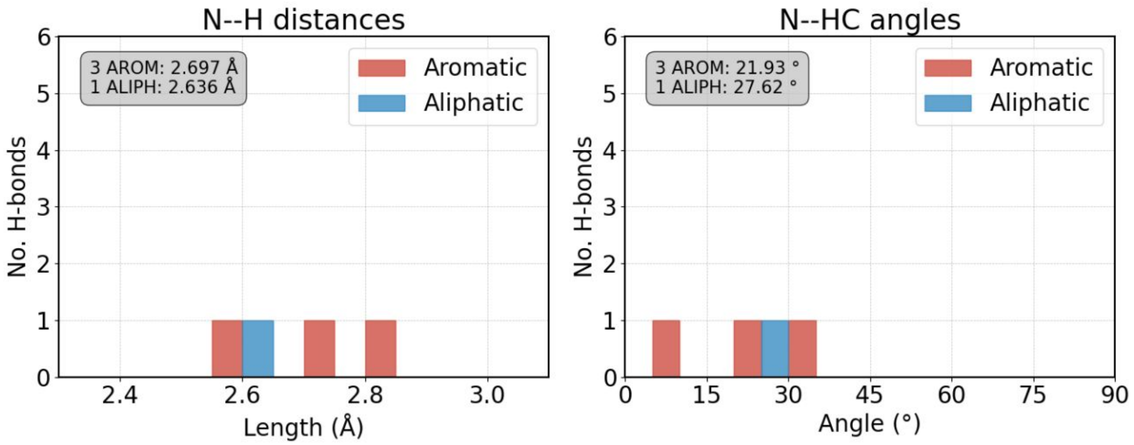

A_2

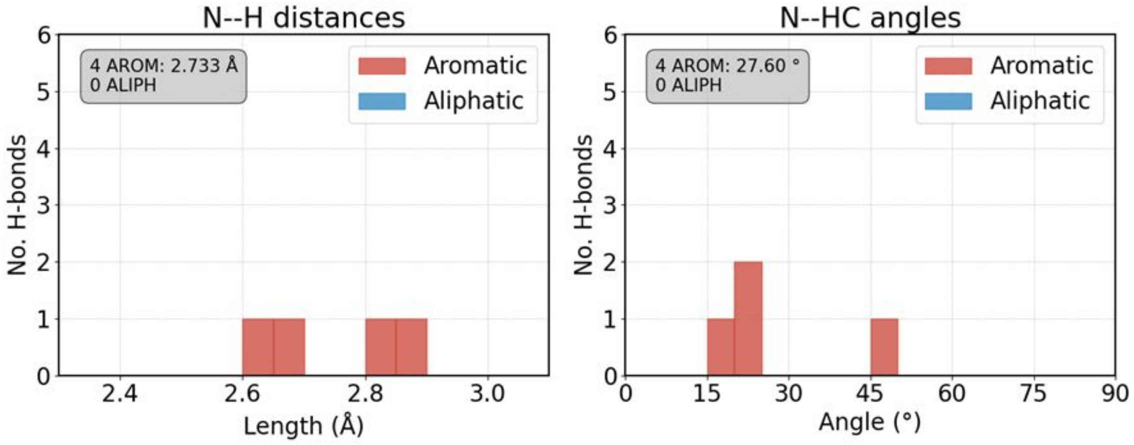

B_1

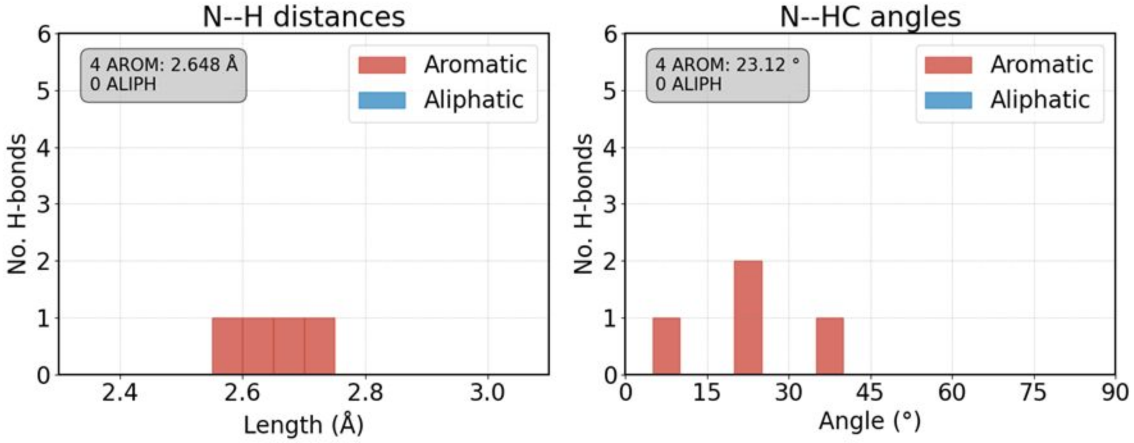

B_2

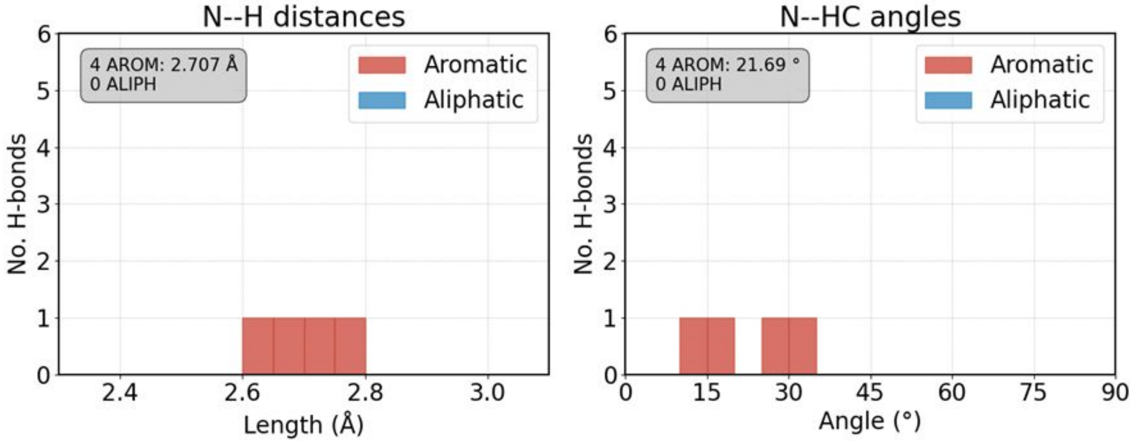

Figure S8. Averaged distributions of N--H distances and N--CH angles in the bulk crystalline cells of DCV-1. The mean values for both aromatic and aliphatic bond types are presented in the grey panel.

DCV-2

A

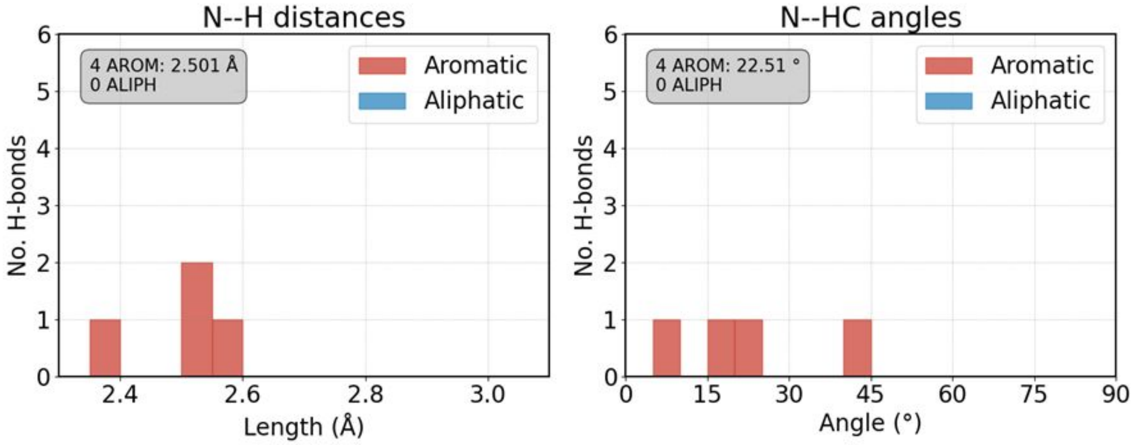
N--H distances
4 AROM: 2.501 Å
0 ALIPH
Aromatic
Aliphatic
No. H-bonds
Length (Å)
N--HC angles
4 AROM: 22.51 °
0 ALIPH
Aromatic
Aliphatic
No. H-bonds
Angle (°)

B

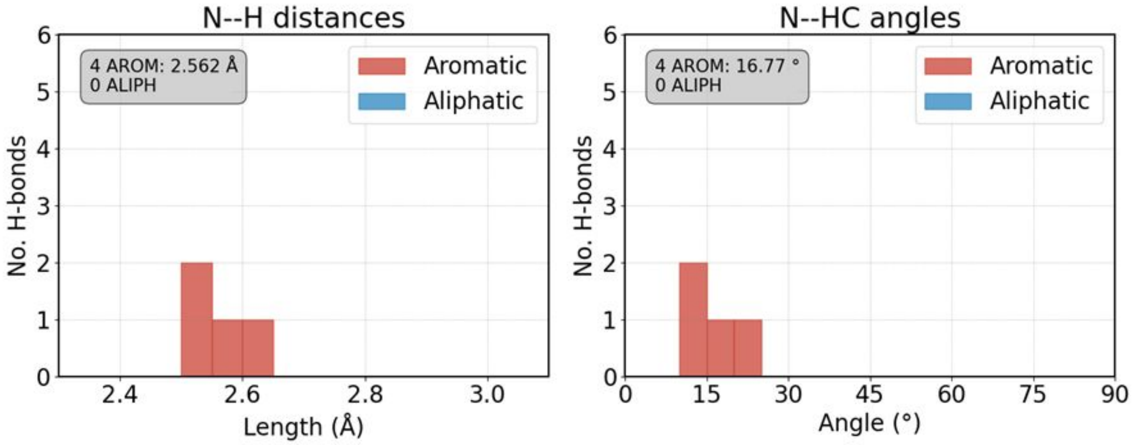
N--H distances
4 AROM: 2.562 Å
0 ALIPH
Aromatic
Aliphatic
No. H-bonds
Length (Å)
N--HC angles
4 AROM: 16.77 °
0 ALIPH
Aromatic
Aliphatic
No. H-bonds
Angle (°)

C

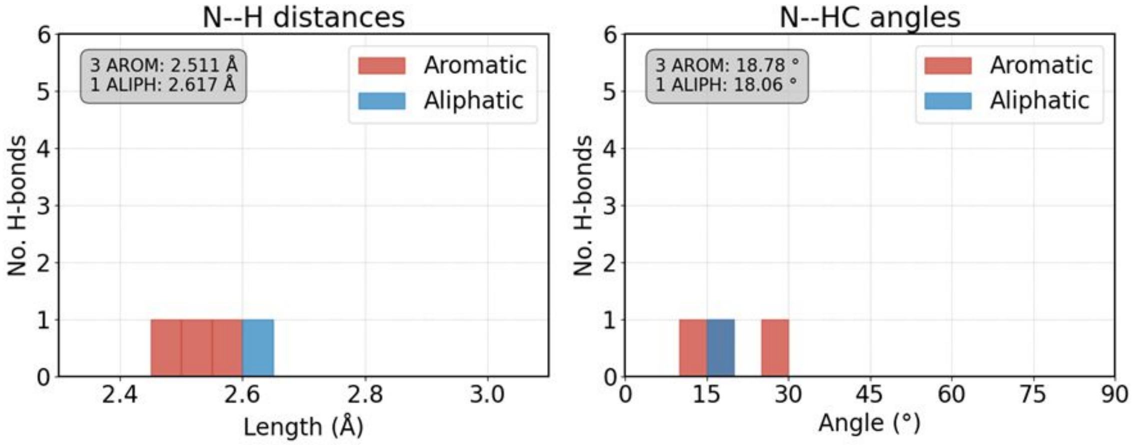

Figure S9. Averaged distributions of N--H distances and N--CH angles in the bulk crystalline cells of DCV-2. The mean values for both aromatic and aliphatic bond types are presented in the grey panel.

DCV-3

A_1

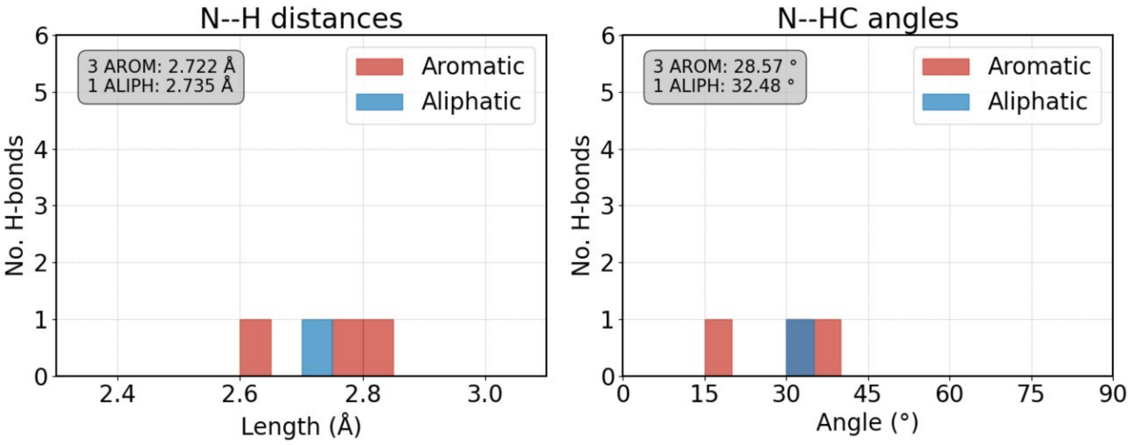
N--H distances
3 AROM: 2.722 Å
1 ALIPH: 2.735 Å
Aromatic
Aliphatic
No. H-bonds
Length (Å)
N--HC angles
3 AROM: 28.57 °
1 ALIPH: 32.48 °
Aromatic
Aliphatic
No. H-bonds
Angle (°)

A_2

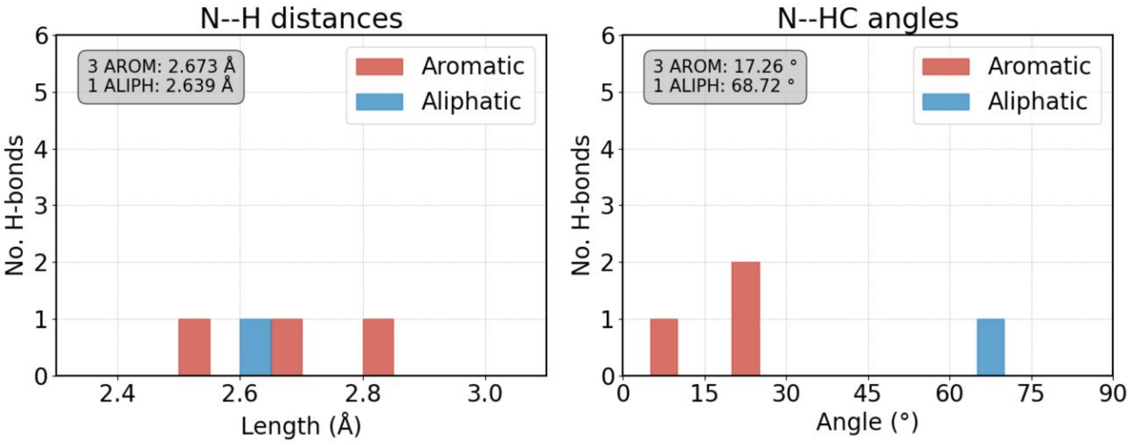
N--H distances
3 AROM: 2.673 Å
1 ALIPH: 2.639 Å
Aromatic
Aliphatic
No. H-bonds
Length (Å)
N--HC angles
3 AROM: 17.26 °
1 ALIPH: 68.72 °
Aromatic
Aliphatic
No. H-bonds
Angle (°)

B_1

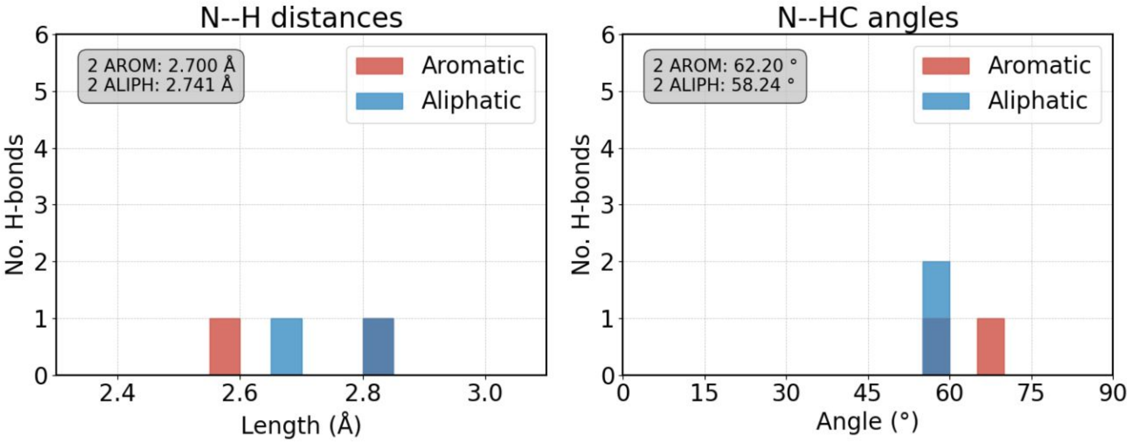
N--H distances
2 AROM: 2.700 Å
2 ALIPH: 2.741 Å
Aromatic
Aliphatic
No. H-bonds
Length (Å)
N--HC angles
2 AROM: 62.20 °
2 ALIPH: 58.24 °
Aromatic
Aliphatic
No. H-bonds
Angle (°)

B_2

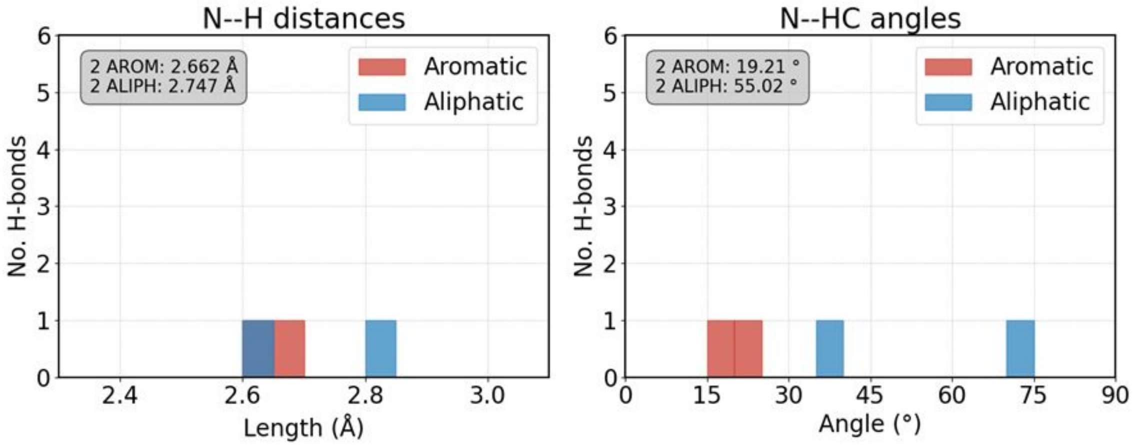

Figure S10. Averaged distributions of N--H distances and N--CH angles in the bulk crystalline cells of DCV-3. The mean values for both aromatic and aliphatic bond types are presented in the grey panel.

References

(1) Comer, J.; Gumbart, J. C.; Hénin, J.; Lelièvre, T.; Pohorille, A.; Chipot, C. The Adaptive Biasing Force Method: Everything You Always Wanted To Know but Were Afraid To Ask. *J. Phys. Chem. B* **2015**, *119* (3), 1129–1151. https://doi.org/10.1021/jp506633n.

(2) Horowitz, G.; Bachet, B.; Yassar, A.; Lang, P.; Demanze, F.; Fave, J.-L.; Garnier, F. Growth and Characterization of Sexithiophene Single Crystals. *Chem. Mater.* **1995**, *7* (7), 1337–1341. https://doi.org/10.1021/cm00055a010.

(3) Siegrist, T.; Fleming, R. M.; Haddon, R. C.; Laudise, R. A.; Lovinger, A. J.; Katz, H. E.; Bridenbaugh, P.; Davis, D. D. The Crystal Structure of the High-Temperature Polymorph of α–Hexathienyl (α–6T/HT). *J. Mater. Res.* **1995**, *10* (9), 2170–2173. https://doi.org/10.1557/JMR.1995.2170.

(4) Fitzner, R.; Mena-Osteritz, E.; Mishra, A.; Schulz, G.; Reinold, E.; Weil, M.; Körner, C.; Ziehlke, H.; Elschner, C.; Leo, K.; Riede, M.; Pfeiffer, M.; Uhrich, C.; Bäuerle, P. Correlation of π-Conjugated Oligomer Structure with Film Morphology and Organic Solar Cell Performance. *J. Am. Chem. Soc.* **2012**, *134* (27), 11064–11067. https://doi.org/10.1021/ja302320c.

(5) Gudovannyy, A. O.; Schäfer, J. M.; Gerdes, O.; Hildebrandt, D.; Mattersteig, G.; Pfeiffer, M.; Ortmann, F. Predicting 2D Crystal Packing in Thin Films of Small Molecule Organic Materials. *Adv. Funct. Mater.* **2025**, 2421048. https://doi.org/10.1002/adfm.202421048.
